# Psoriasis and Cardiovascular Comorbidities: Focusing on Severe Vascular Events, Cardiovascular Risk Factors and Implications for Treatment

**DOI:** 10.3390/ijms18102211

**Published:** 2017-10-21

**Authors:** Stephen Chu-Sung Hu, Cheng-Che E. Lan

**Affiliations:** 1 Department of Dermatology, College of Medicine, Kaohsiung Medical University, Kaohsiung 807, Taiwan; stephenhu30@hotmail.com; 2 Department of Dermatology, Kaohsiung Medical University Hospital, Kaohsiung 807, Taiwan

**Keywords:** psoriasis, cardiovascular disease, cerebrovascular disease, atherosclerosis, hypertension, diabetes mellitus, obesity, dyslipidemia, metabolic syndrome, systemic inflammation

## Abstract

Psoriasis is a common and chronic inflammatory disease of the skin. It may impair the physical and psychosocial function of patients and lead to decreased quality of life. Traditionally, psoriasis has been regarded as a disease affecting only the skin and joints. More recently, studies have shown that psoriasis is a systemic inflammatory disorder which can be associated with various comorbidities. In particular, psoriasis is associated with an increased risk of developing severe vascular events such as myocardial infarction and stroke. In addition, the prevalence rates of cardiovascular risk factors are increased, including hypertension, diabetes mellitus, dyslipidemia, obesity, and metabolic syndrome. Consequently, mortality rates have been found to be increased and life expectancy decreased in patients with psoriasis, as compared to the general population. Various studies have also shown that systemic treatments for psoriasis, including methotrexate and tumor necrosis factor-α inhibitors, may significantly decrease cardiovascular risk. Mechanistically, the presence of common inflammatory pathways, secretion of adipokines, insulin resistance, angiogenesis, oxidative stress, microparticles, and hypercoagulability may explain the association between psoriasis and cardiometabolic disorders. In this article, we review the evidence regarding the association between psoriasis and cardiovascular comorbidities, focusing on severe vascular events, cardiovascular risk factors and implications for treatment.

## 1. Introduction

Psoriasis is a common and chronic inflammatory disease, and may cause significant impairment to the patient’s quality of life [1,2]. Traditionally, psoriasis has been regarded as a disease affecting only the skin and joints. In recent years, studies from different countries have shown that psoriasis is a systemic inflammatory disease, which is often associated with various comorbidities. In particular, there is a greater risk of developing severe vascular events such as cardiovascular and cerebrovascular diseases [3,4,5,6,7]. In addition, the prevalence rates of cardiovascular risk factors are increased in psoriasis patients, including hypertension, diabetes, obesity, dyslipidemia, subclinical atherosclerosis, and smoking [8,9,10]. It has been proposed that systemic inflammation may provide a mechanistic link between psoriasis and cardiometabolic disorders.

Some studies have also investigated the relationship between the severity of psoriasis and the risk of cardiovascular comorbidities. The definition of severe psoriasis varies in different studies. In some studies (particularly large-scale epidemiological studies), patients were classified as severe psoriasis if they required systemic therapy (including methotrexate, retinoid, cyclosporine, biological agents, or phototherapy) [3,11,12]. In other studies, severe psoriasis was defined in terms of Psoriasis Area and Severity Index score (for example, PASI > 15) [13], or body surface area involvement (for example, BSA > 10%) [14,15].

Previous studies have shown that mortality rates are increased in psoriasis patients compared to healthy controls [16,17,18], and the life expectancy of patients with moderate to severe psoriasis is decreased by approximately 5 years, mainly due to cardiovascular comorbidities [19]. Furthermore, the presence of cardiovascular comorbidities in patients with psoriasis has been found to be associated with greatly increased economic and healthcare burden [20,21]. Therefore, physicians should be aware of the cardiovascular risk in patients with psoriasis, and administer appropriate treatments to prevent the future development of vascular events.

## 2. Psoriasis and Severe Vascular Events

A large number of epidemiological studies performed in various countries have demonstrated that psoriasis is associated with increased prevalence of cardiovascular diseases [22,23,24]. A large-scale population-based epidemiological study performed in the United Kingdom using the General Practice Research Database demonstrated that the risk of myocardial infarction is increased in patients with psoriasis [3]. Moreover, there was an association between the risk of myocardial infarction and psoriasis disease severity. The relative risk was greater in younger patients, but the risk was still significantly increased in elderly patients who were 60 years of age. Another population-based cohort study performed in the United Kingdom found increased risk of major adverse cardiovascular events (including myocardial infarction, stroke and cardiovascular mortality) in patients with psoriasis [25]. Epidemiological studies in the United States and Canada have also demonstrated that psoriasis patients have a higher risk of developing myocardial infarction [26,27,28,29]. Population-based studies performed in Denmark found that the risk of myocardial infarction is increased in patients with severe psoriasis but not mild psoriasis [12]. A population-based study in Taiwan found an increased risk of myocardial infarction in patients with psoriasis [30]. An epidemiological study in Japan also showed an association between psoriasis and coronary heart disease [31]. In addition, a cohort study from the United Kingdom revealed that the life expectancy of patients with severe psoriasis is reduced by about 6 years, mainly as a result of cardiovascular mortality [32]. On the other hand, a few studies in certain populations found no significant association between psoriasis and risk of cardiovascular disease [33,34,35].

Moreover, patients with psoriasis were shown to have an increased risk of developing cerebrovascular disease (stroke), which correlates with the severity of psoriasis disease [8,11,24,36,37,38,39,40,41,42]. On the other hand, some studies found no significant association between psoriasis and cerebrovascular disease [27,43,44,45]. These discrepancies in findings may be due to differences in the study population and the methodology used. A recent meta-analysis found that the risk of stroke (expressed in terms of the hazard ratio) was 1.10 and 1.38 for mild and severe psoriasis, respectively, and the risk of myocardial infarction (expressed in terms of the hazard ratio) was 1.20 and 1.70 for mild and severe psoriasis, respectively [46]. Another meta-analysis found that the risk ratios for stroke were 1.12 for mild psoriasis and 1.56 for severe psoriasis [24]. The findings from studies investigating the risk of severe vascular events (including myocardial infarction, cerebrovascular disease, and cardiovascular death) in patients with psoriasis are summarized in Table 1.

We have previously performed a population-based study using information from the Taiwanese National Health Insurance Database. The study involved 8180 patients with psoriasis and 163,600 controls. The time relationship between the development of psoriasis and metabolic disorders (hypertension, diabetes or dyslipidemia) was investigated. Psoriasis was regarded as the initiator of the systemic inflammatory march if the metabolic disorder appeared after the diagnosis of psoriasis. On the other hand, psoriasis was regarded as the amplifier of the inflammatory march if the metabolic disorder appeared before the diagnosis of psoriasis. It was found that the risk of developing severe vascular events is higher when psoriasis acts as the disease amplifier compared to when it acts as the disease initiator. In addition, when psoriasis acts as the disease amplifier, methotrexate therapy was associated with a lower risk of developing cerebrovascular disease [50].

In addition, the presence of concomitant psychological disorders may also increase the risk of cardiovascular disease in psoriasis patients. A population-based cohort study in Taiwan found that the risks of ischemic heart disease and stroke are higher in psoriasis patients with sleep disorders compared to psoriasis patients without sleep disorders [51]. In addition, it is known that the psoriasis patients have a higher prevalence of depression [52,53], and the presence of depression has been shown to further increase the risk of myocardial infarction, stroke and cardiovascular mortality in patients with psoriasis [54].

We have also previously performed a nationwide population-based study using the Taiwanese National Health Insurance Database to explore the effect of anxiety on the risk of developing cerebrovascular disease in patients with psoriasis. Our results showed that the prevalence of anxiety disorder is higher in patients with psoriasis compared to non-psoriatic controls, and psoriasis patients with anxiety have a higher risk of developing cerebrovascular disease compared to psoriasis patients without anxiety (hazard ratio 1.37) [55].

## 3. Psoriasis and Atherosclerosis

A number of studies have attempted to determine the causal relationship between psoriasis and cardiovascular disease. Atherosclerosis is the major pathological change preceding the development of myocardial infarction and stroke. Patients with psoriasis have been found to have increased arterial stiffness compared to healthy controls, and there is a positive correlation between arterial stiffness and psoriasis disease duration [56,57]. Atherosclerosis may also develop following chronic vascular inflammation. Using positron emission tomography/computed tomography (PET/CT), it has been found that patients with psoriasis have greater aortic vascular inflammation, and there is an association between the severity of psoriasis and the degree of vascular inflammation [58]. In addition, it has been shown that improvement of psoriasis skin disease can lead to a reduction of aortic vascular inflammation [59].

Coronary artery atherosclerosis is an important risk factor for ischemic heart disease. Various studies have found that patients with psoriasis have increased prevalence and severity of coronary artery calcification and atherosclerosis (measured by cardiac computed tomography, coronary computed tomography angiography, or coronary angiography) compared to healthy controls [60,61,62,63,64,65,66]. Moreover, a reduction in psoriasis disease severity has been found to be associated with an improvement in coronary atherosclerosis [67]. The development of coronary atherosclerosis in psoriasis patients may be partially related to impaired cholesterol efflux capacity from macrophages [68,69].

Atherosclerosis of the carotid artery is recognized as a risk factor for the development of cerebrovascular disease. Using carotid artery ultrasound, various studies have found that patients with psoriasis have greater carotid intima-media thickness compared to healthy controls, indicating carotid atherosclerosis [70,71,72,73,74]. The severity of skin disease (measured by Psoriasis Area and Severity Index (PASI) score) has been found to correlate with carotid intima-media thickness values [75]. A recent meta-analysis of 20 studies also confirmed that patients with psoriasis have greater carotid intima-media thickness values and decreased flow-mediated dilation compared to healthy controls, thus demonstrating an association between psoriasis and subclinical carotid atherosclerosis [76].

In addition, epicardial fat tissue has been shown to be a cardiovascular risk factor and is associated with the development of atherosclerosis. The epicardial fat thickness or area (measured by transthoracic echocardiography or computed tomography) has been found to be greater in patients with psoriasis compared to healthy controls [77,78,79,80]. This may contribute to the greater cardiovascular risk in patients with psoriasis [81].

## 4. Psoriasis and Cardiovascular Risk Factors

Various studies have found that psoriasis is associated with greater prevalence of cardiovascular risk factors, including hypertension, diabetes mellitus, dyslipidemia, obesity, and the metabolic syndrome [8,10]. These cardiovascular risk factors will be discussed in more detail below.

### 4.1. Hypertension

A number of studies have shown significant associations between psoriasis and the prevalence of hypertension [8,43,82,83,84,85]. A meta-analysis found increased prevalence of hypertension in psoriasis patients, with odds ratios of 1.30 for mild psoriasis and 1.49 for severe psoriasis [86]. Patients with psoriasis were also found to have higher risk of uncontrolled hypertension, and the risk correlates with psoriasis disease severity [15].

In addition, the presence of hypertension may increase the risk of incident psoriasis. In the Nurses’ Health Study involving 77,728 women, patients with hypertension were found to have a higher risk of developing psoriasis [87]. This may be associated with the use of beta-blockers to treat hypertension.

### 4.2. Diabetes Mellitus

A number of studies have found that the prevalence rate of diabetes mellitus is higher in patients with psoriasis [8,43,85,88,89]. According to a previous meta-analysis, psoriasis is associated with a greater risk of diabetes mellitus with odds ratios of 1.53 for mild psoriasis and 1.97 for severe psoriasis [90]. In a population-based cohort study from Taiwan, it was found that psoriasis is associated with the development of diabetes, and the risk is greater for severe disease (hazard ratio 2.06) compared to mild disease (hazard ratio 1.28) [91]. Patients with psoriasis have also been found to have greater insulin resistance compared to normal controls, suggesting that psoriasis may be a prediabetic disorder [92]. In patients with psoriasis associated with diabetes, glycemic control with hypoglycemic medications has been shown to improve the skin condition [93].

Metformin is known as an insulin sensitizer and has been demonstrated to prevent the development of diabetes, promote weight loss, and reduce the incidence of metabolic syndrome, which may lead to a more favorable cardiovascular risk profile [94,95,96,97,98]. Previously, treatment of psoriasis patients with metabolic disorders with metformin has been demonstrated to decrease psoriasis severity and improve the parameters of the metabolic syndrome [99,100]. In patients with diabetes, treatment with thiazolidinediones and metformin has also been shown to reduce the risk of developing psoriasis [101,102], while regular insulin use may increase psoriasis risk [102].

The risk of diabetic complications in patients with psoriasis has also been investigated. Patients with concomitant diabetes and psoriasis have been shown to have greater risk of developing microvascular and macrovascular complications, compared to diabetic patients without psoriasis [103]. Patients with severe psoriasis have also been found to have greater levels of advanced glycation end products in their blood and skin [104].

### 4.3. Dyslipidemia

A number of studies have demonstrated a higher prevalence of dyslipidemia in patients with psoriasis [9,10,43,105,106,107]. Patients with psoriasis have been found to have increased risk of hypercholesterolemia [108,109,110]. In addition, a number of studies have shown that psoriasis is associated with decreased high-density lipoprotein (HDL) level and increased low-density lipoprotein (LDL) level [107,110,111,112]. Abnormalities in lipoprotein profile have also been found in pediatric patients with psoriasis [113].

Apart from blood cholesterol abnormalities, serum triglyceride levels have also been found to be increased in patients with psoriasis compared to healthy controls [107,110]. In addition, serum fatty acid profile was found to be abnormal in patients with psoriasis compared to healthy controls [114].

Some studies have also shown that dyslipidemia may precede the development of psoriasis. The Nurses’ Health Study II showed that patients with hypercholesterolemia have increased risk of developing psoriasis (hazard ratio 1.25), and the risk is greater for patients with longer duration of hypercholesterolemia (more than 7 years) [115]. Therefore, the temporal relationship between psoriasis and dyslipidemia requires further investigation.

### 4.4. Obesity

It is known that psoriasis is associated with increased prevalence of obesity [43,106,110,116,117], particularly central obesity (as measured by waist-to-height ratio or waist circumference) [118,119]. In a cross-sectional survey performed in Germany, the mean body mass index (BMI) of patients with psoriasis (28.0) was shown to be significantly higher than normal controls (25.9) [120]. The severity of psoriatic skin disease had also been shown to correlate with the degree of obesity (determined by BMI or waist-to-height ratio) [118,121]. The association between psoriasis and obesity (particularly central adiposity) has also been found in the pediatric population [122,123,124,125].

The temporal relationship between psoriasis and obesity is currently not completely defined. Various studies have indicated that obesity may be a risk factor for developing psoriasis [126]. A previous large-scale prospective study found that increased BMI is associated with greater incidence of psoriasis, which indicates that obesity occurs before the development of psoriasis [116]. In addition, a dose-response relationship was found between the degree of obesity (BMI) and the risk of developing psoriasis [116,127]. On the other hand, another study demonstrated that obesity occurred after the onset of psoriasis, suggesting that obesity is a consequence and not a risk factor for psoriasis [128].

Various studies have also shown that in psoriasis patients with obesity, weight loss (including dietary measures, physical exercise or surgical gastrectomy) can lead to improvement of the skin condition [129,130,131,132,133,134]. In patients with psoriasis who were obese, weight loss has also been shown to increase the efficacy of anti-TNF-α biologic therapy [135].

The molecular mechanisms underlying the association between psoriasis and obesity are currently not clearly understood. Various studies have shown that the disordered production of adipokines from fat tissue in obese patients with psoriasis may lead to chronic skin and systemic inflammation and increased cardiovascular risk [136,137].

### 4.5. Metabolic Syndrome

Components of the metabolic syndrome include hyperglycemia, central/abdominal obesity, hypertension, and dyslipidemia (hypertriglyceridemia or low HDL level) [138,139]. Various studies have shown that psoriasis is associated with an increased prevalence of the metabolic syndrome [43,106,140,141,142,143]. Moreover, the frequency of the metabolic syndrome was found to increase with greater psoriasis disease severity [14,144,145]. An association between psoriasis and metabolic syndrome has also been found in the Asian population, including Thailand [146], China [147], and India [148]. In addition, children with psoriasis were shown to have a higher prevalence of the metabolic syndrome [124,149].

### 4.6. Cigarette Smoking and Alcohol Consumption

Both cigarette smoking and alcohol use are known cardiovascular risk factors. Various studies have shown increased prevalence of cigarette smoking and alcohol consumption in patients with psoriasis [128,150,151,152,153,154,155,156,157]. A recent meta-analysis also demonstrated a significant association between smoking and psoriasis disease severity [158]. However, it is not clearly defined whether cigarette smoking and alcohol consumption may increase the risk of developing psoriasis, or whether they occur as a consequence of psoriasis-associated psychological stress. A previous meta-analysis indicated that cigarette smoking predates psoriasis and may be an independent risk factor for psoriasis development [152]. Mechanistically, previous studies have demonstrated increased amount of peripheral blood Th17 cells in psoriasis patients who smoke [159], which may partially explain the increased risk of psoriasis in cigarette smokers.

## 5. Relationship between Severity of Psoriasis (in Terms of PASI and BSA) and Cardiovascular Risk

Some of the studies showing an association between the severity of psoriasis and the risk of cardiovascular comorbidities have used the PASI score as a measure of psoriasis severity [58,59,67,118,144,160,161,162]. On the other hand, some studies have shown an association between BSA involvement and cardiovascular risk [14,15,125,163,164]. Currently, it is not known whether PASI or BSA correlate better with cardiovascular risk in patients with psoriasis. In addition, at present there is no specific PASI/BSA threshold above which systemic therapy is recommended due to increased risk of cardiovascular comorbidities. Although patients with palmoplantar or pustular psoriasis may have low PASI/BSA scores, they may have significant systemic inflammation. A few small-scale studies have shown an association between palmoplantar/nail and pustular forms of psoriasis and cardiovascular comorbidities [165,166], although this is contradicted by another study [167].

## 6. Psoriatic Arthritis and Cardiovascular Comorbidities

A number of studies have demonstrated that psoriatic arthritis is associated with higher prevalence of cardiovascular and metabolic diseases. In particular, there is a greater risk of developing severe vascular events such as myocardial infarction and stroke [25,168,169,170,171]. Patients with psoriatic arthritis have also been found to have higher risk of atherosclerosis [172,173]. In addition, the prevalence of cardiovascular risk factors are increased in patients with psoriatic arthritis, including hypertension, diabetes, obesity, dyslipidemia, and metabolic syndrome [174,175,176,177,178].

We have previously carried out a retrospective cohort study using the Taiwanese National Health Insurance Database. In total, 284 psoriasis patients with arthritis and 7648 psoriasis patients without arthritis were included. It was found that psoriasis patients with arthritis had a higher risk of developing severe vascular events (hazard ratio 1.46 for cardiovascular disease and 1.82 for cerebrovascular disease), compared to psoriasis patients without arthritis [179].

## 7. Pathogenic Mechanisms

Various studies have attempted to define the molecular mechanisms responsible for the association between psoriasis and cardiovascular comorbidities. Mechanisms which have been proposed include shared genetic factors, common inflammatory pathways, secretion of adipokines, insulin resistance, lipoprotein composition and function, angiogenesis, oxidative stress, microparticles, and hypercoagulability.

### 7.1. Shared Genetic Factors

One particular question is whether there is a genetic basis for the increased prevalence of cardiovascular comorbidities in psoriasis patients. Genome-wide association studies have found increased inheritance of certain genes that are associated with cardiovascular risk in patients with psoriasis, indicating shared genetic factors [180]. Other shared genetic factors between psoriasis and cardiometabolic diseases that have been found include CDKAL1, apolipoprotein E, and interleukins [181,182,183]. However, a number of studies have shown that psoriasis and cardiometabolic disorders do not share common genetic risk loci [184,185].

### 7.2. Common Inflammatory Pathways

The association between psoriasis and cardiovascular disease may be partly explained by common inflammatory pathways [186,187]. Chronic skin inflammation may lead to vascular and systemic inflammation, atherosclerosis, and thrombosis. Patients with psoriasis have been shown to have increased vascular, subcutaneous and hepatic inflammation (assessed by PET/CT scan) compared to healthy controls [188,189,190,191]. Animal studies have also shown that chronic skin inflammation may induce vascular inflammation [192]. Moreover, the systemic inflammatory marker C-reactive protein (CRP) has been found to be elevated in patients with psoriasis [193,194]. Furthermore, a previous meta-analysis of 5 microarray data sets found that psoriasis lesional skin shows increased expression of genes associated with atherosclerosis signaling and fatty acid metabolism [195].

The cytokine profiles of psoriasis skin lesions and atherosclerosis vascular lesions are very similar, showing increased number of Th1 and Th17 lymphocytes [186,196,197]. The Th1 and Th17 cytokine pathways have been shown to be involved in the pathogenesis of both psoriasis and atherosclerosis [197,198,199,200,201,202]. Similar to psoriasis, patients with ischemic heart disease have increased levels of Th17-related cytokines (IL-17, IL-6 and IL-8) in their peripheral blood [203]. Therefore, the overexpression of Th17 cytokines in patients with psoriasis may mediate vascular inflammation and the development of atherosclerosis and cardiovascular comorbidities [204,205]. In addition, the skin lesions and blood of psoriasis patients have been shown to contain increased levels of cardiovascular biomarkers, including monocyte chemoattractant protein-1 and macrophage-derived chemokine [206].

However, the temporal relationship between psoriatic systemic inflammation and cardiovascular disease remains unclear. It is possible that the systemic inflammation of psoriasis may lead to the development of cardiovascular diseases, or alternatively the cardiovascular risk factors may cause immune dysfunction leading to psoriasis. In the theory known as the “psoriatic march”, it is proposed that psoriasis may induce systemic inflammation leading to insulin resistance, endothelial dysfunction, and development of atherosclerosis and cardiovascular comorbidities [196,207].

### 7.3. Adipokines

The systemic inflammation associated with psoriasis may lead to inflammation of the adipose tissue, which may promote the release of pro-inflammatory adipokines into the peripheral blood. A number of studies have shown that adipokines (including leptin, adiponectin and resistin) may be involved in the development of the metabolic syndrome and cardiovascular comorbidities [208].

Leptin is known as a pro-inflammatory adipokine. Previously, serum leptin levels have been shown to be elevated in psoriasis patients and correlate with psoriasis disease severity [209,210,211]. Moreover, increased leptin expression may induce increased levels of pro-inflammatory cytokines leading to exacerbation of psoriasis [137,212]. In patients with psoriasis, increased leptin expression has been shown to be associated with the metabolic syndrome [209,213]. Serum leptin level has also been found to correlate with subclinical atherosclerosis (carotid intima-media wall thickness) in psoriasis patients [214]. On the other hand, adiponectin is an anti-inflammatory adipokine. Previous studies have found decreased serum levels of adiponectin in psoriasis patients [211,215], which may contribute to the systemic inflammation of psoriasis. In addition, resistin and visfatin are pro-inflammatory adipokines. Serum levels of resistin and visfatin have been found to be increased in psoriasis patients and correlate with disease severity [214,216,217].

### 7.4. Insulin Resistance

It has been shown that patients with psoriasis have greater insulin resistance compared to normal controls [92]. The development of insulin resistance in psoriasis patients may provide a mechanistic explanation for the increased risk of atherosclerosis and cardiovascular comorbidities.

### 7.5. Lipoprotein Composition and Function

The composition and function of lipoprotein particles may be altered in psoriasis patients. Previous studies have shown that the antioxidant and anti-inflammatory activities of HDL are reduced in patients with psoriasis compared to healthy controls, indicating functional impairment of HDL in psoriasis [218]. In patients with psoriasis, changes in the composition of HDL particles may also impede their capacity to induce cholesterol efflux from macrophages, thus leading to atherosclerosis and cardiovascular disease [68,69]. Interestingly, both the HDL particle composition and cholesterol efflux ability become normalized following anti-psoriasis therapy [219].

### 7.6. Angiogenesis and Oxidative Stress

Both psoriasis skin lesions and atherosclerotic plaques are characterized by increased angiogenesis [220,221,222]. The production of pro-angiogenic factors (including vascular endothelial growth factor and interleukin-8) from psoriasis plaques may lead to the development and progression of atherosclerosis [223,224]. In addition, common oxidative stress signaling pathways may underlie the association between psoriasis and atherosclerosis [223,225,226].

### 7.7. Microparticles

Microparticles are composed of plasma membrane vesicles which may be derived from endothelial cells, platelets and monocytes/macrophages. They contain nucleic acids and various inflammatory mediators [7]. They may be released as a result of cell activation or apoptosis, and are involved in the development of atherosclerosis [227]. Previous studies have shown that psoriasis patients have increased blood levels of microparticles, which may contribute to the development of atherosclerosis and cardiovascular comorbidities [228,229,230].

### 7.8. Hypercoagulability

Patients with psoriasis have been shown to exhibit increased platelet activation and aggregation [229,231,232]. Moreover, psoriasis patients may have increased blood levels of plasminogen activator inhibitor-1 [233]. These factors may lead to a hypercoagulable state and increased risk of thromboembolic events in patients with psoriasis.

### 7.9. Serum Homocysteine Level

Homocysteine induces oxidative stress, and elevated plasma homocysteine level has been found to be associated with the development of atherosclerosis and cardiovascular diseases [234,235]. Previous studies have shown that plasma homocysteine levels are elevated and folate levels decreased in patients with psoriasis, and correlate with psoriasis disease severity [236]. This may contribute to the formation of atherosclerotic plaques in psoriasis patients.

## 8. Biomarkers of Systemic Inflammation

Although there appears to be an association between psoriasis severity and the risk of cardiovascular diseases, the severity of skin disease may not closely correlate with the degree of vascular or systemic inflammation. Patients with psoriatic arthritis may show only mild skin disease, but may have severe systemic inflammation. Therefore, identification of serum biomarkers of systemic inflammation is important for assessing cardiovascular risk [7].

Psoriasis patients have been found to have altered levels of traditional cardiovascular biomarkers, including CRP, soluble CD40 ligand, human matrix Gla protein and fetuin-A [194,237,238,239]. Other proposed biomarkers of inflammation and cardiovascular risk in psoriasis include serum YKL-40 [240], GlycA [241], and complement C3 [242]. The peripheral blood neutrophil/lymphocyte ratio has also been found to be elevated in psoriasis patients and may act as a marker of subclinical atherosclerosis [243,244]. In addition, serum myeloperoxidase (MPO) level is elevated in psoriasis and may be a biomarker for cardiovascular risk [245].

## 9. Effects of Anti-Psoriasis Therapies on Cardiovascular Comorbidities

Adequate treatment of psoriasis patients, particularly those with moderate to severe disease, may decrease the risk of cardiovascular comorbidities. This may occur as a result of suppression of systemic inflammation. Systemic forms of treatment for psoriasis include ultraviolet light phototherapy, immunosuppressive agents (methotrexate, cyclosporine), oral retinoid (acitretin), fumaric acid esters, and biological agents (TNF-α inhibitors) [246,247]. Various treatment modalities for psoriasis may have different impacts on the cardiovascular system. A number of studies have shown that phototherapy does not have a significant impact on the risk of cardiovascular events [248,249,250]. Systemic treatments for psoriasis and their effects on cardiovascular risk are discussed below. The findings from studies investigating the effects of different psoriasis treatments on the risk of cardiovascular disease in patients with psoriasis are summarized in Table 2.

### 9.1. Methotrexate

A number of studies have demonstrated that treatment of psoriasis patients with methotrexate may decrease the risk of cardiovascular events (including ischemic heart disease, stroke, and cardiovascular death) [248,250,251,252,253,254]. In particular, low-dose methotrexate has been found to be effective in decreasing the risk of cardiovascular diseases in patients with psoriasis [251].

We have previously performed a population-based study utilizing the Taiwanese National Health Insurance Database. A total of 8180 patients with psoriasis and 163,600 patients without psoriasis were included. It was found that psoriasis is associated with greater risk of cerebrovascular disease (hazard ratio 1.28 compared to non-psoriasis patients). This risk was significantly reduced with methotrexate treatment (hazard ratio 0.50) but not retinoid treatment. In addition, this protective effect was only seen with low cumulative methotrexate dose but not high cumulative dose [48]. Therefore, low-dose methotrexate treatment is effective in preventing the occurrence of cerebrovascular disease in psoriasis patients. In addition, we have previously found that methotrexate treatment was associated with lower risk of cardiovascular disease in psoriasis patients without arthritis [179].

### 9.2. Cyclosporine

In a subset of patients with psoriasis, treatment with cyclosporine may lead to impaired renal function, hypertension and increased cardiovascular risk. In addition, hyperlipidemia may occur in psoriasis patients treated with cyclosporine. Therefore, it is recommended that in patients with psoriasis, cyclosporine should only be used for a limited duration, and another systemic agent should be substituted once the skin condition is improved [7]. A previous study has shown that treatment of patients with severe psoriasis with cyclosporine does not have a protective effect on the development of cardiovascular events (myocardial infarction, stroke, cardiovascular death) [252].

### 9.3. Acitretin

Acitretin is most commonly used to treat generalized pustular psoriasis. In a subset of patients, acitretin may cause hyperlipidemia, which should be actively treated to avoid increased cardiovascular risk. Previous studies in humans and animals have shown that retinoids may improve and ameliorate the formation of atherosclerotic plaques [255,256,257,258,259,260,261]. Further investigations are required to determine whether treatment of psoriasis patients with retinoids may prevent the development of atherosclerosis and decrease the risk of cardiovascular events.

### 9.4. Fumaric Acid Esters

Fumaric acid esters have been widely used in Germany to treat psoriasis. Treatment with fumaric acid esters have been shown to decrease serum CRP level and increase adiponectin level (a cardioprotective adipokine) in patients with psoriasis [268,269]. Further investigations are required to determine whether this form of treatment may reduce cardiovascular risk.

### 9.5. Tumor Necrosis Factor-Alpha (TNF-α) Inhibitors

Various studies have shown that treatment of psoriasis patients with TNF-α inhibitors may lower the risk of cardiovascular comorbidities. Compared to patients who received topical therapy, psoriasis patients treated with TNF-α inhibitors showed a significant decrease in the risk of myocardial infarction (hazard ratio 0.50) [263]. Other studies have also confirmed the beneficial effects of TNF-α inhibitors in reducing the risk of myocardial infarction in psoriasis patients [264,265,266,270,271,272]. In addition, a number of studies have shown that treatment of psoriasis patients with TNF-α inhibitors leads to decreased risk of cardiovascular events compared to methotrexate, and greater risk reduction was found with longer duration of anti-TNF-α treatment [267,273].

Treatment of psoriasis patients with TNF-α inhibitors may also have beneficial effects on the development of atherosclerosis and components of the metabolic syndrome. Anti-TNF-α therapy in patients with severe psoriasis has been shown to improve subclinical cardiovascular disease (abnormalities in echocardiogram) [13], improve coronary microvascular function (determined by transthoracic Doppler echocardiography) [274], and attenuate the progression in coronary artery disease (assessed by coronary computed tomography) [275]. Treatment of patients with moderate to severe psoriasis with adalimumab for 6 months led to improvement in endothelial function (brachial artery reactivity) and carotid arterial stiffness [276]. However, another study found that treatment of patients with psoriasis with adalimumab for 16 weeks had no significant effect on vascular inflammation (determined by PET/CT scan) in the carotid arteries or aorta [277]. In addition, TNF-α inhibitor treatment may decrease the risk of diabetes mellitus in patients with psoriasis [278], and improve insulin sensitivity both in diabetic [279] and non-diabetic patients [280]. However, another study found that combination anti-TNF-α and methotrexate treatment did not improve hemoglobin A_1C_ and fasting glucose level compared to methotrexate treatment alone [281].

On the other hand, a number of studies have shown that treatment with TNF-α inhibitors may lead to increased body weight and BMI in patients with psoriasis [282,283,284]. Therefore, body weight should be monitored in psoriasis patients treated with TNF-α inhibitors, and appropriate weight reduction measures may be required to reduce the cardiovascular risk.

Furthermore, treatment of psoriasis patients with TNF-α inhibitors may improve biomarkers of cardiovascular risk. Patients with psoriasis who were treated with etanercept showed decreased blood levels of cardiovascular biomarkers, such as soluble VCAM-1, soluble ICAM-1, soluble E-selectin, MMP-9, MPO, and tPAI-1 [249]. Psoriasis patients who received adalimumab also showed decreased serum levels of cardiovascular biomarkers E-selectin and IL-22 [285,286]. Other studies demonstrated that treatment of psoriasis patients with etanercept led to decreased CRP levels [287,288]. Treatment of psoriasis patients with TNF-α inhibitors has been shown to suppress serum leptin levels [289]. Furthermore, patients with psoriasis who received anti-TNF-α agents showed decreased amount of peripheral blood endothelial and platelet microparticles [290].

### 9.6. Anti-IL-12/23 Agents

Two meta-analysis studies have suggested that short-term treatment of psoriasis patients with anti-IL-12/23 agents (ustekinumab and briakinumab) may be associated with increased risk of major adverse cardiovascular events [291,292]. On the other hand, a long-term study over 5 years indicated that ustekinumab treatment does not increase or decrease the incidence of major adverse cardiovascular events in patients with psoriasis [293]. Other studies have also shown that treatment of psoriasis patients with ustekinumab does not lead to increased risk of major adverse cardiovascular events [248,250,294,295].

A recent study showed that ustekinumab may improve coronary and myocardial function in psoriasis patients [296], while another study revealed that it may increase fasting sugar and triglyceride levels [297]. Further investigations are required to confirm whether ustekinumab may have beneficial or detrimental effects on the cardiovascular system in the long term.

### 9.7. Anti-IL-17 Agents

Anti-IL-17 agents (such as secukinumab) have recently been used for the treatment of psoriasis. Patients with psoriasis treated with anti-IL-17 agents have shown no increased incidence of major adverse cardiovascular events [298,299]. However, further long-term studies are needed to evaluate whether anti-IL-17 agents may have beneficial effects on cardiovascular comorbidities in patients with psoriasis [300].

### 9.8. Apremilast

Apremilast is an oral phosphodiesterase-4 inhibitor which has recently been approved for the treatment of psoriasis [301]. In randomized controlled trials, patients with psoriasis receiving apremilast have shown no increased risk of major cardiac events up to 156 weeks [302,303]. Further long-term large-scale studies are required to determine whether this relatively new drug may have beneficial effects on cardiovascular risk in psoriasis.

## 10. Conclusions

Recent studies have shown that psoriasis is not a disease affecting only the skin and joints, but may be associated with various cardiovascular and metabolic disorders. Patients with psoriasis have been shown to have a higher prevalence of cardiovascular risk factors including hypertension, diabetes, dyslipidemia, obesity, metabolic syndrome and smoking, and are at increased risk of developing severe vascular events (including myocardial infarction and stroke). The presence of common inflammatory pathways may provide an explanation for the association between psoriasis and cardiovascular comorbidities.

Physicians should be more aware of the cardiovascular risk when assessing patients with psoriasis. In particular, adequate treatment of psoriasis may not only ameliorate the skin condition, but also decrease the risk and severity of cardiovascular and metabolic disorders. Further investigations are required to clarify the mechanisms underlying the association between psoriasis and cardiovascular comorbidities, and define optimal treatment regimens to reduce the risk of cardiovascular events in patients with psoriasis.

## Figures and Tables

**Table 1:** Studies investigating the risk of severe vascular events (including myocardial infarction, cerebrovascular disease, and cardiovascular death) in patients with psoriasis.

Study	Cardiovascular Comorbidities	Number of Patients/Controls	Relative Risk	Population/Type of Study
Abuabara et al., 2010 [32]	Cardiovascular death	Severe psoriasis: 3603; Controls: 14,330	Hazard ratio: 1.57 (95% CI 1.26–1.96)	United Kingdom/Cohort study
Ahlehoff et al., 2011 [41]	Composite endpoint (myocardial infarction, stroke and cardiovascular death)	Mild psoriasis: 34,371; Severe psoriasis: 2621; Controls: 4,003,265	Rate ratio: Composite endpoint: Mild psoriasis: 1.20 (95% CI 1.14–1.25); Severe psoriasis: 1.58 (95% CI 1.36–1.82); Stroke: Mild psoriasis: 1.25 (95% CI 1.16–1.33); Severe psoriasis: 1.71 (95% CI 1.39–2.11); Myocardial infarction: Mild psoriasis: 1.22 (95% CI 1.12–1.33); Severe psoriasis: 1.45 (95% CI 1.10–1.90); Cardiovascular death: Mild psoriasis: 1.14 (95% CI 1.06–1.22); Severe psoriasis 1.57 (95% CI 1.27–1.94)	Denmark/Cohort study
Ahlehoff et al., 2011 [47]	Composite cardiovascular endpoint (recurrent myocardial infarction, stroke and cardiovascular death) after first time myocardial infarction	Patients with first time myocardial infarction; Psoriasis: 462; Controls: 48,935	Hazard ratio: 1.26 (95% CI 1.06–1.54)	Denmark/Cohort study
Ahlehoff et al., 2012 [40]	Ischemic stroke	Mild psoriasis: 36,765; Severe psoriasis: 2793; Controls: 4,478,926	Rate ratio: Age < 50 years: Mild psoriasis: 1.97 (95% CI 1.66–2.34); Severe psoriasis: 2.80 (95% CI 1.81–4.34); Age ≥ 50 years: Mild psoriasis: 1.13 (95% CI 1.04–1.21); Severe psoriasis: 1.34 (95% CI 1.04–1.71)	Denmark/Cohort study
Brauchli et al., 2009 [44]	Myocardial infarction, stroke or transient ischemic attack	Psoriasis: 36,702; Controls: 36,702	Odds ratio: Myocardial infarction: Overall: 1.14 (95% CI 0.93–1.41); Age < 60 years: 1.66 (95% CI 1.03–2.66); Age ≥ 60 years: 0.99 (95% CI 0.77–1.26); Stroke: Overall: 0.93 (95% CI 0.77–1.13); Age < 60 years: 0.52 (95% CI 0.29–0.93); Age ≥ 60 years: 0.99 (95% CI 0.80–1.22); Transient ischemic attack: Overall: 1.00 (95% CI 0.81–1.25); Age < 60 years: 1.28 (95% CI 0.61–2.68); Age ≥ 60 years: 1.02 (95% CI 0.80–1.29)	United Kingdom/Inception cohort study with nested case-control analysis
Chiang et al., 2012 [39]	Ischemic stroke	Psoriasis: 2783; Controls: 13,910	Hazard ratio: 1.27 (95% CI 1.05–1.52)	Taiwan/Retrospective cohort study
Dowlatshahi et al., 2013 [35]	Cardiovascular disease (coronary heart disease, stroke, heart failure)	Psoriasis: 262; Controls: 8009	Hazard ratio: 0.73 (95% CI 0.50–1.06)	Netherlands/Prospective cohort study
Dregan et al., 2014 [45]	Coronary heart disease Stroke	Severe psoriasis: 5648; Mild psoriasis: 85,232; Controls: 373,851	Hazard ratio: Mild psoriasis: Stroke: 1.08 (95% CI 0.98–1.18); Coronary heart disease: 1.03 (95% CI 0.97–1.11); Severe psoriasis: Stroke: 0.93 (95% CI 0.64–1.36); Coronary heart disease: 1.29 (95% CI 1.01–1.64)	United Kingdom/Cohort study
Egeberg et al., 2017 [12]	Myocardial infarction	Mild psoriasis: 49,646; Severe psoriasis: 11,957; Controls: 4,300,085	Hazard ratio: Mild psoriasis: 1.02 (95% CI 0.96–1.09); Severe psoriasis: 1.21 (95% CI 1.07–1.37)	Denmark/Cohort study
Gelfand et al., 2006 [3]	Myocardial infarction	Mild psoriasis: 127,139; Severe psoriasis: 3837; Controls: 556,995	Relative risk: 30-year-old: Mild psoriasis: 1.29 (95% CI 1.14–1.46); Severe psoriasis: 3.10 (95% CI 1.98–4.86); 60-year-old: Mild psoriasis: 1.08 (95% CI 1.03–1.13); Severe psoriasis: 1.36 (95% CI 1.13–1.64)	United Kingdom/Prospective cohort study
Gelfand et al., 2009 [11]	Stroke	Mild psoriasis: 129,143 (controls: 496,666); Severe psoriasis: 3603 (controls 14,330)	Hazard ratio: Mild psoriasis: 1.06 (95% CI 1.0–1.1); Severe psoriasis: 1.43 (95% CI 1.1–1.9)	United Kingdom/Cohort study
Kaye et al., 2008 [8]	Myocardial infarction Stroke	Psoriasis: 44,164; Controls: 219,784	Hazard ratio: Myocardial infarction: 1.21 (95% CI 1.10–1.32); Stroke: 1.12 (95% CI 1.00–1.25)	United Kingdom/Cohort study
Lai et al., 2016 [27]	Myocardial infarction Ischemic heart disease Stroke	Psoriasis: 520; Total subjects: 19,065	Odds ratio: Myocardial infarction: 2.24 (95% CI 1.27–3.95); Ischemic heart disease: 1.90 (95% CI 1.18–3.05); Stroke: 1.01 (95% CI 0.48–2.16)	United States/Cross-sectional study
Lan et al., 2012 [48]	Cerebrovascular disease	Psoriasis: 8180; Controls: 163,600	Hazard ratio: 1.28 (95% CI 1.162–1.413)	Taiwan/Retrospective cohort study
Levesque et al., 2013 [29]	Myocardial infarction	Psoriasis: 31,421; Controls: 31,421	Hazard ratio: 1.17 (95% CI 1.04–1.31)	Canada/Retrospective cohort study
Li et al., 2012 [28]	Nonfatal cardiovascular disease (nonfatal myocardial infarction, nonfatal stroke)	Participants: 96,008 (women); Psoriasis: 2463	Hazard ratio: Myocardial infarction: 1.70 (95% CI 1.01–2.86); Stroke: 1.45 (95% CI 0.80–2.65)	United States/Cohort study
Lin et al., 2011 [30]	Myocardial infarction	Psoriasis: 4752; Controls: 23,760	Hazard ratio: 2.10 (95% CI 1.27–3.43)	Taiwan/Retrospective cohort study
Mallbris et al., 2004 [49]	Cardiovascular mortality	Psoriasis inpatients: 8991; Psoriasis outpatients: 19,757	Standardized mortality ratio: Inpatients: 1.52 (95% CI 1.44–1.60); Outpatients: 0.94 (95% CI 0.89–0.99)	Sweden/Cohort study
Mehta et al., 2010 [18]	Cardiovascular mortality	Severe psoriasis: 3603; Controls: 14,330	Hazard ratio: 1.57 (95% CI 1.26–1.96)	United Kingdom/Cohort study
Ogdie et al., 2015 [25]	Major adverse cardiovascular events (including myocardial infarction, cerebrovascular accidents and cardiovascular death)	Psoriasis: 138,424; Controls: 81,573	Hazard ratio: Mild psoriasis (no DMARD): 1.08 (95% CI 1.02–1.15); Severe psoriasis (DMARD user): 1.42 (95% CI 1.17–1.73)	United Kingdom/Cohort study
Prodanovich et al., 2009 [42]	Ischemic heart disease; Cerebrovascular disease; Peripheral vascular disease	Psoriasis: 3236; Controls: 2500	Odds ratio: Ischemic heart disease: 1.78 (95% CI 1.51–2.11); Cerebrovascular disease: 1.70 (95% CI 1.33–2.17); Peripheral vascular disease: 1.98 (95% CI 1.38–2.82)	United States/Observational cross-sectional study
Shiba et al., 2016 [31]	Coronary heart disease	Hospital-based population: 113,065; Psoriasis: 1197	Odds ratio: 1.27 (95% CI 1.01–1.58)	Japan/Cross-sectional study
Stern et al., 2011 [33]	Cardiovascular mortality	Severe psoriasis: 1376	Standard mortality ratio: 1.02 (95% CI 0.90–1.16)	United States/Prospective cohort study
Wakkee et al., 2010 [34]	Ischemic heart disease hospitalization	Psoriasis: 15,820; Controls: 27,577	Hazard ratio: 1.05 (95% CI 0.95–1.17)	Netherlands/Cohort study
Wu et al., 2015 [26]	Myocardial infarction	Mild psoriasis: 10,173 (controls: 50,865); Severe psoriasis: 3841 (controls: 19,205)	Hazard ratio: Mild psoriasis: 1.31 (95% CI 1.14–1.51); Severe psoriasis: 1.28 (95% CI 1.02–1.60)	United States/Retrospective cohort study

CI = confidence interval.

**Table 2:** Studies investigating the effects of different psoriasis treatments on the risk of cardiovascular disease in patients with psoriasis.

Study	Treatment for Psoriasis	Cardiovascular Endpoint	Number of Patients	Relative Risk	Population/Type of Study
Abuabara et al., 2011 [262]	Systemic immunomodulatory therapies (methotrexate, cyclosporine, alefacept, efalizumab, adalimumab, etanercept, infliximab)	Myocardial infarction	Psoriasis: 25,554; Phototherapy: 4220; Systemic treatment: 20,094; Both treatments: 1240	Hazard ratio (compared to UVB phototherapy): 1.33 (95% CI 0.90–1.96)	United States/Cohort study
Ahlehoff et al., 2013 [254]	Biological agents; Methotrexate	Cardiovascular death, myocardial infarction and stroke	Severe psoriasis: 2400; Biological agents: 693; Methotrexate: 799; Other therapies: 908	Hazard ratio (compared to other therapies): Biological agents: 0.48 (95% CI 0.17–1.38); Methotrexate: 0.50 (95% CI 0.26–0.97)	Denmark/Retrospective cohort study
Ahlehoff et al., 2015 [252]	Methotrexate; Cyclosporine; Retinoids; TNF-α inhibitors; Ustekinumab	Cardiovascular events (cardiovascular death, myocardial infarction, stroke)	Severe psoriasis: 6902; Methotrexate: 3564; Cyclosporine: 244; Retinoids: 756; TNF-α inhibitors: 959; Ustekinumab: 178	Hazard ratio (compared to other therapies): Methotrexate: 0.53 (95% CI 0.34–0.83); Cyclosporine: 1.06 (95% CI 0.26–4.27); Retinoids: 1.80 (95% CI 1.03–2.96); TNF-α inhibitors: 0.46 (95% CI 0.22–0.98); Ustekinumab: 1.52 (95% CI 0.47–4.94)	Denmark/Cohort study
Chin et al., 2013 [179]	Methotrexate; Retinoid	Cardiovascular disease; Cerebrovascular disease	Psoriasis patients without arthritis: 7648	Hazard ratio (compared to no methotrexate and no retinoid treatment): Cardiovascular disease: Methotrexate: 0.39 (95% CI 0.20–0.76); Retinoid 0.47 (95% CI 0.26–0.83); Cerebrovascular disease: Methotrexate: 0.42 (95% CI 0.19–0.95); Retinoid: 0.67 (95% CI 0.35–1.31)	Taiwan/Retrospective cohort study
Lan et al., 2012 [48]	Methotrexate; Retinoid	Cerebrovascular disease	Psoriasis: 8180; Methotrexate: 258; Retinoid: 193	Hazard ratio (compared to no methotrexate and no retinoid treatment): Methotrexate: 0.50 (95% CI 0.27–0.92); Retinoid: 0.70 (95% CI 0.39–1.23)	Taiwan/Retrospective cohort study
Prodanovich et al., 2005 [251]	Methotrexate	Vascular disease (including cardiovascular disease, cerebrovascular disease, atherosclerosis)	Psoriasis: 7615	Relative risk (compared to no methotrexate treatment): Methotrexate: 0.73 (95% CI 0.55–0.98); Low cumulative dose methotrexate: 0.50 (95% CI 0.31–0.79)	United States/Retrospective cohort study
Wu et al., 2012 [263]	TNF inhibitor	Myocardial infarction	Psoriasis: 8845; TNF inhibitor: 1673	Hazard ratio (compared to topical therapy): TNF inhibitor: 0.50 (95% CI 0.32–0.79)	United States/Retrospective cohort study
Wu et al., 2013 [264]	TNF inhibitor; Oral/phototherapy	Myocardial infarction	Psoriasis: 8845; Caucasians: 4645 (TNF inhibitor: 857; Oral/phototherapy: 1011; Topical: 2777); Non-Caucasians: 4200 (TNF inhibitor: 816; Oral/phototherapy: 1086; Topical: 2298)	Hazard ratio (compared to topical therapy): Caucasians: TNF inhibitors: 0.35 (95% CI 0.20–0.62); Oral/phototherapy: 0.36 (95% CI 0.22–0.59); Non-Caucasians: TNF inhibitors: 0.27 (95% CI 0.11–0.67); Oral/phototherapy: 0.58 (95% CI 0.32–1.04)	United States/Retrospective cohort study
Wu et al., 2013 [265]	TNF inhibitor (etanercept or monoclonal antibody)	Myocardial infarction	Etanercept: 976; Monoclonal antibody: 217; Topical therapy: 5075	Hazard ratio (compared to topical agents): Etanercept: 0.53 (95% CI 0.31–0.92); Monoclonal antibody: 0.25 (95% CI 0.06–1.03)	United States/Retrospective cohort study
Wu et al., 2014 [266]	TNF inhibitor	Myocardial infarction	Psoriasis (treated with TNF inhibitor): 846; Psoriasis (not treated with TNF inhibitor): 7172	Hazard ratio (compared to psoriasis patients not treated with TNF inhibitors): 0.26 (95% CI 0.12–0.56)	United States/Retrospective cohort study
Wu et al., 2017 [267]	TNF inhibitor	Major cardiovascular events (myocardial infarction, stroke or transient ischemic attack, unstable angina)	TNF inhibitor: 9148; Methotrexate: 8581	Hazard ratio (compared to methotrexate): Major cardiovascular event: 0.55 (95% CI 0.45–0.67) Myocardial infarction: 0.49 (95% CI 0.34–0.71); Stroke or TIA: 0.55 (95% CI 0.42–0.71); Unstable angina: 0.58 (95% CI 0.41–0.82)	United States/Retrospective cohort study

CI = confidence interval.

## Abbreviations

PET/CT	Positron emission tomography/computed tomography
PASI	Psoriasis area and severity index
BSA	Body surface area
HDL	High-density lipoprotein
LDL	Low-density lipoprotein
BMI	Body mass index
CRP	C-Reactive protein
TNF-α	Tumor necrosis factor-α

## References

[1] Augustin M., Radtke M.A. (2014). Quality of life in psoriasis patients. Expert Rev. Pharmacoecon. Outcomes Res..

[2] Quintard B., Constant A., Bouyssou-Gauthier M.L., Paul C., Truchetet F., Thomas P., Guiguen Y., Taieb A. (2011). Validation of a specific health-related quality of life instrument in a large cohort of patients with psoriasis: The QualiPso Questionnaire. Acta Derm. Venereol..

[3] Gelfand J.M., Neimann A.L., Shin D.B., Wang X., Margolis D.J., Troxel A.B. (2006). Risk of myocardial infarction in patients with psoriasis. JAMA.

[4] Furue M., Tsuji G., Chiba T., Kadono T. (2017). Cardiovascular and Metabolic diseases comorbid with psoriasis: Beyond the skin. Intern. Med..

[5] Shahwan K.T., Kimball A.B. (2015). Psoriasis and cardiovascular disease. Med. Clin. N. Am..

[6] Takeshita J., Grewal S., Langan S.M., Mehta N.N., Ogdie A., van Voorhees A.S., Gelfand J.M. (2017). Psoriasis and comorbid diseases: Epidemiology. J. Am. Acad. Dermatol..

[7] Ryan C., Kirby B. (2015). Psoriasis is a systemic disease with multiple cardiovascular and metabolic comorbidities. Dermatol. Clin..

[8] Kaye J.A., Li L., Jick S.S. (2008). Incidence of risk factors for myocardial infarction and other vascular diseases in patients with psoriasis. Br. J. Dermatol..

[9] Shah K., Mellars L., Changolkar A., Feldman S.R. (2017). Real-world burden of comorbidities in US patients with psoriasis. J. Am. Acad. Dermatol..

[10] Ma L., Li M., Wang H., Li Y., Bai B. (2014). High prevalence of cardiovascular risk factors in patients with moderate or severe psoriasis in northern China. Arch. Dermatol. Res..

[11] Gelfand J.M., Dommasch E.D., Shin D.B., Azfar R.S., Kurd S.K., Wang X., Troxel A.B. (2009). The risk of stroke in patients with psoriasis. J. Investig. Dermatol..

[12] Egeberg A., Thyssen J.P., Jensen P., Gislason G.H., Skov L. (2017). Risk of myocardial infarction in patients with psoriasis and psoriatic arthritis: A nationwide cohort study. Acta Derm. Venereol..

[13] Heredi E., Vegh J., Pogacsas L., Gaspar K., Varga J., Kincse G., Zeher M., Szegedi A., Gaal J. (2016). Subclinical cardiovascular disease and it’s improvement after long-term TNF-alpha inhibitor therapy in severe psoriatic patients. J. Eur. Acad. Dermatol. Venereol. JEADV.

[14] Langan S.M., Seminara N.M., Shin D.B., Troxel A.B., Kimmel S.E., Mehta N.N., Margolis D.J., Gelfand J.M. (2012). Prevalence of metabolic syndrome in patients with psoriasis: A population-based study in the United Kingdom. J. Investig. Dermatol..

[15] Takeshita J., Wang S., Shin D.B., Mehta N.N., Kimmel S.E., Margolis D.J., Troxel A.B., Gelfand J.M. (2015). Effect of psoriasis severity on hypertension control: A population-based study in the United Kingdom. JAMA Dermatol..

[16] Masson W., Rossi E., Galimberti M.L., Krauss J., Navarro Estrada J., Galimberti R., Cagide A. (2017). Mortality in patients with psoriasis. A retrospective cohort study. Med. Clin..

[17] Gelfand J.M., Troxel A.B., Lewis J.D., Kurd S.K., Shin D.B., Wang X., Margolis D.J., Strom B.L. (2007). The risk of mortality in patients with psoriasis: Results from a population-based study. Arch. Dermatol..

[18] Mehta N.N., Azfar R.S., Shin D.B., Neimann A.L., Troxel A.B., Gelfand J.M. (2010). Patients with severe psoriasis are at increased risk of cardiovascular mortality: Cohort study using the General Practice Research Database. Eur. Heart J..

[19] Siegel D., Devaraj S., Mitra A., Raychaudhuri S.P., Raychaudhuri S.K., Jialal I. (2013). Inflammation, atherosclerosis, and psoriasis. Clin. Rev. Allergy Immunol..

[20] Augustin M., Vietri J., Tian H., Gilloteau I. (2017). Incremental burden of cardiovascular comorbidity and psoriatic arthritis among adults with moderate-to-severe psoriasis in five European countries. J. Eur. Acad. Dermatol. Venereol. JEADV.

[21] Feldman S.R., Tian H., Gilloteau I., Mollon P., Shu M. (2017). Economic burden of comorbidities in psoriasis patients in the United States: Results from a retrospective U.S. database. BMC Health Serv. Res..

[22] Benson M.M., Frishman W.H. (2015). The heartbreak of psoriasis: A review of cardiovascular risk in patients with psoriasis. Cardiol. Rev..

[23] Coumbe A.G., Pritzker M.R., Duprez D.A. (2014). Cardiovascular risk and psoriasis: Beyond the traditional risk factors. Am. J. Med..

[24] Armstrong E.J., Harskamp C.T., Armstrong A.W. (2013). Psoriasis and major adverse cardiovascular events: A systematic review and meta-analysis of observational studies. J. Am. Heart Assoc..

[25] Ogdie A., Yu Y., Haynes K., Love T.J., Maliha S., Jiang Y., Troxel A.B., Hennessy S., Kimmel S.E., Margolis D.J. (2015). Risk of major cardiovascular events in patients with psoriatic arthritis, psoriasis and rheumatoid arthritis: A population-based cohort study. Ann. Rheum. Dis..

[26] Wu J.J., Choi Y.M., Bebchuk J.D. (2015). Risk of myocardial infarction in psoriasis patients: A retrospective cohort study. J. Dermatol. Treat..

[27] Lai Y.C., Yew Y.W. (2016). Psoriasis as an Independent Risk Factor for Cardiovascular Disease: An Epidemiologic Analysis Using a National Database. J. Cutan. Med. Surg..

[28] Li W.Q., Han J.L., Manson J.E., Rimm E.B., Rexrode K.M., Curhan G.C., Qureshi A.A. (2012). Psoriasis and risk of nonfatal cardiovascular disease in U.S. women: A cohort study. Br. J. Dermatol..

[29] Levesque A., Lachaine J., Bissonnette R. (2013). Risk of myocardial infarction in canadian patients with psoriasis: A retrospective cohort study. J. Cutan. Med. Surg..

[30] Lin H.W., Wang K.H., Lin H.C., Lin H.C. (2011). Increased risk of acute myocardial infarction in patients with psoriasis: A 5-year population-based study in Taiwan. J. Am. Acad. Dermatol..

[31] Shiba M., Kato T., Funasako M., Nakane E., Miyamoto S., Izumi T., Haruna T., Inoko M. (2016). Association between psoriasis vulgaris and coronary heart disease in a hospital-based population in Japan. PLoS ONE.

[32] Abuabara K., Azfar R.S., Shin D.B., Neimann A.L., Troxel A.B., Gelfand J.M. (2010). Cause-specific mortality in patients with severe psoriasis: A population-based cohort study in the U.K. Br. J. Dermatol..

[33] Stern R.S., Huibregtse A. (2011). Very severe psoriasis is associated with increased noncardiovascular mortality but not with increased cardiovascular risk. J. Investig. Dermatol..

[34] Wakkee M., Herings R.M., Nijsten T. (2010). Psoriasis may not be an independent risk factor for acute ischemic heart disease hospitalizations: Results of a large population-based Dutch cohort. J. Investig. Dermatol..

[35] Dowlatshahi E.A., Kavousi M., Nijsten T., Ikram M.A., Hofman A., Franco O.H., Wakkee M. (2013). Psoriasis is not associated with atherosclerosis and incident cardiovascular events: The Rotterdam Study. J. Investig. Dermatol..

[36] Ahlehoff O., Gislason G., Lamberts M., Folke F., Lindhardsen J., Larsen C.T., Torp-Pedersen C., Hansen P.R. (2015). Risk of thromboembolism and fatal stroke in patients with psoriasis and nonvalvular atrial fibrillation: A Danish nationwide cohort study. J. Intern. Med..

[37] Samarasekera E.J., Neilson J.M., Warren R.B., Parnham J., Smith C.H. (2013). Incidence of cardiovascular disease in individuals with psoriasis: A systematic review and meta-analysis. J. Investig. Dermatol..

[38] Xu T., Zhang Y.H. (2012). Association of psoriasis with stroke and myocardial infarction: Meta-analysis of cohort studies. Br. J. Dermatol..

[39] Chiang C.H., Huang C.C., Chan W.L., Huang P.H., Chen Y.C., Chen T.J., Chung C.M., Lin S.J., Chen J.W., Leu H.B. (2012). Psoriasis and increased risk of ischemic stroke in Taiwan: A nationwide study. J. Dermatol..

[40] Ahlehoff O., Gislason G.H., Jorgensen C.H., Lindhardsen J., Charlot M., Olesen J.B., Abildstrom S.Z., Skov L., Torp-Pedersen C., Hansen P.R. (2012). Psoriasis and risk of atrial fibrillation and ischaemic stroke: A Danish Nationwide Cohort Study. Eur. Heart J..

[41] Ahlehoff O., Gislason G.H., Charlot M., Jorgensen C.H., Lindhardsen J., Olesen J.B., Abildstrom S.Z., Skov L., Torp-Pedersen C., Hansen P.R. (2011). Psoriasis is associated with clinically significant cardiovascular risk: A Danish nationwide cohort study. J. Intern. Med..

[42] Prodanovich S., Kirsner R.S., Kravetz J.D., Ma F., Martinez L., Federman D.G. (2009). Association of psoriasis with coronary artery, cerebrovascular, and peripheral vascular diseases and mortality. Arch. Dermatol..

[43] Miller I.M., Ellervik C., Yazdanyar S., Jemec G.B. (2013). Meta-analysis of psoriasis, cardiovascular disease, and associated risk factors. J. Am. Acad. Dermatol..

[44] Brauchli Y.B., Jick S.S., Miret M., Meier C.R. (2009). Psoriasis and risk of incident myocardial infarction, stroke or transient ischaemic attack: An inception cohort study with a nested case-control analysis. Br. J. Dermatol..

[45] Dregan A., Charlton J., Chowienczyk P., Gulliford M.C. (2014). Chronic inflammatory disorders and risk of type 2 diabetes mellitus, coronary heart disease, and stroke: A population-based cohort study. Circulation.

[46] Raaby L., Ahlehoff O., de Thurah A. (2017). Psoriasis and cardiovascular events: Updating the evidence. Arch. Dermatol. Res..

[47] Ahlehoff O., Gislason G.H., Lindhardsen J., Olesen J.B., Charlot M., Skov L., Torp-Pedersen C., Hansen P.R. (2011). Prognosis following first-time myocardial infarction in patients with psoriasis: A Danish nationwide cohort study. J. Intern. Med..

[48] Lan C.C., Ko Y.C., Yu H.S., Wu C.S., Li W.C., Lu Y.W., Chen Y.C., Chin Y.Y., Yang Y.H., Chen G.S. (2012). Methotrexate reduces the occurrence of cerebrovascular events among Taiwanese psoriatic patients: A nationwide population-based study. Acta Derm. Venereol..

[49] Mallbris L., Akre O., Granath F., Yin L., Lindelof B., Ekbom A., Stahle-Backdahl M. (2004). Increased risk for cardiovascular mortality in psoriasis inpatients but not in outpatients. Eur. J. Epidemiol..

[50] Su Y.S., Yu H.S., Li W.C., Ko Y.C., Chen G.S., Wu C.S., Lu Y.W., Yang Y.H., Lan C.C. (2013). Psoriasis as initiator or amplifier of the systemic inflammatory march: Impact on development of severe vascular events and implications for treatment strategy. J. Eur. Acad. Dermatol. Venereol. JEADV.

[51] Chiu H.Y., Hsieh C.F., Chiang Y.T., Tsai Y.W., Huang W.F., Li C.Y., Wang T.S., Tsai T.F. (2016). Concomitant sleep disorders significantly increase the risk of cardiovascular disease in patients with psoriasis. PLoS ONE.

[52] Dowlatshahi E.A., Wakkee M., Arends L.R., Nijsten T. (2014). The prevalence and odds of depressive symptoms and clinical depression in psoriasis patients: A systematic review and meta-analysis. J. Investig. Dermatol..

[53] Luca M., Luca A., Musumeci M.L., Fiorentini F., Micali G., Calandra C. (2016). Psychopathological Variables and Sleep Quality in Psoriatic Patients. Int. J. Mol. Sci..

[54] Egeberg A., Khalid U., Gislason G.H., Mallbris L., Skov L., Hansen P.R. (2016). Impact of depression on risk of myocardial infarction, stroke and cardiovascular death in patients with psoriasis: A danish nationwide study. Acta Derm. Venereol..

[55] Lan C.C., Yu H.S., Li W.C., Ko Y.C., Wu C.S., Lu Y.W., Yang Y.H., Chen G.S. (2013). Anxiety contributes to the development of cerebrovascular disease in Taiwanese patients with psoriasis: A population-based study. Eur. J. Dermatol. EJD.

[56] Gisondi P., Fantin F., Del Giglio M., Valbusa F., Marino F., Zamboni M., Girolomoni G. (2009). Chronic plaque psoriasis is associated with increased arterial stiffness. Dermatology.

[57] Choi B.G., Kim M.J., Yang H.S., Lee Y.W., Choe Y.B., Ahn K.J. (2017). Assessment of arterial stiffness in korean patients with psoriasis by cardio-ankle vascular index. Angiology.

[58] Naik H.B., Natarajan B., Stansky E., Ahlman M.A., Teague H., Salahuddin T., Ng Q., Joshi A.A., Krishnamoorthy P., Dave J. (2015). Severity of psoriasis associates with aortic vascular inflammation detected by FDG PET/CT and neutrophil activation in a prospective observational study. Arterioscler. Thromb. Vasc. Biol..

[59] Dey A.K., Joshi A.A., Chaturvedi A., Lerman J.B., Aberra T.M., Rodante J.A., Teague H.L., Harrington C.L., Rivers J.P., Chung J.H. (2017). Association between skin and aortic vascular inflammation in patients with psoriasis: A case-cohort study using positron emission tomography/computed tomography. JAMA Cardiol..

[60] Mansouri B., Kivelevitch D., Natarajan B., Joshi A.A., Ryan C., Benjegerdes K., Schussler J.M., Rader D.J., Reilly M.P., Menter A. (2016). Comparison of coronary artery calcium scores between patients with psoriasis and type 2 diabetes. JAMA Dermatol..

[61] Staniak H.L., Bittencourt M.S., de Souza Santos I., Sharovsky R., Sabbag C., Goulart A.C., Lotufo P.A., Bensenor I.M. (2014). Association between psoriasis and coronary calcium score. Atherosclerosis.

[62] Bissonnette R., Cademartiti F., Maffei E., Tardif J.C. (2017). Increase in coronary atherosclerosis severity and the prevalence of coronary artery mixed plaques in patients with psoriasis. Br. J. Dermatol..

[63] Hjuler K.F., Bottcher M., Vestergaard C., Deleuran M., Raaby L., Botker H.E., Iversen L., Kragballe K. (2015). Increased prevalence of coronary artery disease in severe psoriasis and severe atopic dermatitis. Am. J. Med..

[64] Picard D., Benichou J., Sin C., Abasq C., Houivet E., Koning R., Cribier A., Veber B., Dujardin F., Eltchaninoff H. (2014). Increased prevalence of psoriasis in patients with coronary artery disease: Results from a case-control study. Br. J. Dermatol..

[65] Zeb I., Budoff M. (2015). Coronary artery calcium screening: Does it perform better than other cardiovascular risk stratification tools?. Int. J. Mol. Sci..

[66] Eckert J., Schmidt M., Magedanz A., Voigtlander T., Schmermund A. (2015). Coronary CT angiography in managing atherosclerosis. Int. J. Mol. Sci..

[67] Lerman J.B., Joshi A.A., Chaturvedi A., Aberra T.M., Dey A.K., Rodante J.A., Salahuddin T., Chung J.H., Rana A., Teague H.L. (2017). Coronary plaque characterization in psoriasis reveals high-risk features that improve after treatment in a prospective observational study. Circulation.

[68] Salahuddin T., Natarajan B., Playford M.P., Joshi A.A., Teague H., Masmoudi Y., Selwaness M., Chen M.Y., Bluemke D.A., Mehta N.N. (2015). Cholesterol efflux capacity in humans with psoriasis is inversely related to non-calcified burden of coronary atherosclerosis. Eur. Heart J..

[69] Holzer M., Wolf P., Curcic S., Birner-Gruenberger R., Weger W., Inzinger M., El-Gamal D., Wadsack C., Heinemann A., Marsche G. (2012). Psoriasis alters HDL composition and cholesterol efflux capacity. J. Lipid Res..

[70] Evensen K., Slevolden E., Skagen K., Ronning O.M., Brunborg C., Krogstad A.L., Russell D. (2014). Increased subclinical atherosclerosis in patients with chronic plaque psoriasis. Atherosclerosis.

[71] Santilli S., Kast D.R., Grozdev I., Cao L., Feig R.L., Golden J.B., Debanne S.M., Gilkeson R.C., Orringer C.E., McCormick T.S. (2016). Visualization of atherosclerosis as detected by coronary artery calcium and carotid intima-media thickness reveals significant atherosclerosis in a cross-sectional study of psoriasis patients in a tertiary care center. J. Transl. Med..

[72] Shaharyar S., Warraich H., McEvoy J.W., Oni E., Ali S.S., Karim A., Jamal O., Blaha M.J., Blumenthal R.S., Fialkow J. (2014). Subclinical cardiovascular disease in plaque psoriasis: Association or causal link?. Atherosclerosis.

[73] Uyar B., Akyildiz M., Solak A., Genc B., Saklamaz A. (2015). Relationship between serum fetuin-A levels and carotid intima-media thickness in turkish patients with mild to moderate psoriasis. A case-control study. Acta Dermatovenerol. Croat. ADC.

[74] Steinl D.C., Kaufmann B.A. (2015). Ultrasound imaging for risk assessment in atherosclerosis. Int. J. Mol. Sci..

[75] Banska-Kisiel K., Haberka M., Bergler-Czop B., Brzezinska-Wcislo L., Okopien B., Gasior Z. (2016). Carotid intima-media thickness in patients with mild or moderate psoriasis. Postep. Dermatol. I Alergol..

[76] Fang N., Jiang M., Fan Y. (2016). Association between psoriasis and subclinical atherosclerosis: A meta-analysis. Medicine.

[77] Akyildiz Z.I., Seremet S., Emren V., Ozcelik S., Gediz B., Tastan A., Nazli C. (2014). Epicardial fat thickness is independently associated with psoriasis. Dermatology.

[78] Balci A., Celik M., Balci D.D., Karazincir S., Yonden Z., Korkmaz I., Celik E., Egilmez E. (2014). Patients with psoriasis have an increased amount of epicardial fat tissue. Clin. Exp. Dermatol..

[79] Torres T., Bettencourt N., Mendonca D., Vasconcelos C., Gama V., Silva B.M., Selores M. (2015). Epicardial adipose tissue and coronary artery calcification in psoriasis patients. J. Eur. Acad. Dermatol. Venereol. JEADV.

[80] Wang X., Guo Z., Zhu Z., Bao Y., Yang B. (2016). Epicardial fat tissue in patients with psoriasis:a systematic review and meta-analysis. Lipids Health Dis..

[81] Raposo I., Torres T. (2015). Psoriasis strikes back! Epicardial adipose tissue: Another contributor to the higher cardiovascular risk in psoriasis. Revista Portuguesa de Cardiologia.

[82] Phan C., Sigal M.L., Lhafa M., Barthelemy H., Maccari F., Esteve E., Reguiai Z., Perrot J.L., Chaby G., Maillard H. (2016). Metabolic comorbidities and hypertension in psoriasis patients in France. Comparisons with French national databases. Annales de Dermatologie et de Venereologie.

[83] Armesto S., Coto-Segura P., Osuna C.G., Camblor P.M., Santos-Juanes J. (2012). Psoriasis and hypertension: A case-control study. J. Eur. Acad. Dermatol. Venereol. JEADV.

[84] Cohen A.D., Weitzman D., Dreiher J. (2010). Psoriasis and hypertension: A case-control study. Acta Derm. Venereol..

[85] Qureshi A.A., Choi H.K., Setty A.R., Curhan G.C. (2009). Psoriasis and the risk of diabetes and hypertension: A prospective study of US female nurses. Arch. Dermatol..

[86] Armstrong A.W., Harskamp C.T., Armstrong E.J. (2013). The association between psoriasis and hypertension: A systematic review and meta-analysis of observational studies. J. Hypertens..

[87] Wu S., Han J., Li W.Q., Qureshi A.A. (2014). Hypertension, antihypertensive medication use, and risk of psoriasis. JAMA Dermatol..

[88] Lonnberg A.S., Skov L., Skytthe A., Kyvik K.O., Pedersen O.B., Thomsen S.F. (2016). Association of psoriasis with the risk for type 2 diabetes mellitus and obesity. JAMA Dermatol..

[89] Dubreuil M., Rho Y.H., Man A., Zhu Y., Zhang Y., Love T.J., Ogdie A., Gelfand J.M., Choi H.K. (2014). Diabetes incidence in psoriatic arthritis, psoriasis and rheumatoid arthritis: A UK population-based cohort study. Rheumatology.

[90] Armstrong A.W., Harskamp C.T., Armstrong E.J. (2013). Psoriasis and the risk of diabetes mellitus: A systematic review and meta-analysis. JAMA Dermatol..

[91] Lee M.S., Lin R.Y., Lai M.S. (2014). Increased risk of diabetes mellitus in relation to the severity of psoriasis, concomitant medication, and comorbidity: A nationwide population-based cohort study. J. Am. Acad. Dermatol..

[92] Gyldenlove M., Storgaard H., Holst J.J., Vilsboll T., Knop F.K., Skov L. (2015). Patients with psoriasis are insulin resistant. J. Am. Acad. Dermatol..

[93] Ip W., Kirchhof M.G. (2017). Glycemic control in the treatment of psoriasis. Dermatology.

[94] Glossmann H., Reider N. (2013). A marriage of two “Methusalem” drugs for the treatment of psoriasis?: Arguments for a pilot trial with metformin as add-on for methotrexate. Derm. Endocrinol..

[95] Knowler W.C., Barrett-Connor E., Fowler S.E., Hamman R.F., Lachin J.M., Walker E.A., Nathan D.M. (2002). Reduction in the incidence of type 2 diabetes with lifestyle intervention or metformin. N. Engl. J. Med..

[96] (2012). The 10-year cost-effectiveness of lifestyle intervention or metformin for diabetes prevention: An intent-to-treat analysis of the DPP/DPPOS. Diabetes Care.

[97] Orchard T.J., Temprosa M., Goldberg R., Haffner S., Ratner R., Marcovina S., Fowler S. (2005). The effect of metformin and intensive lifestyle intervention on the metabolic syndrome: The Diabetes Prevention Program randomized trial. Ann. Intern. Med..

[98] (2012). Long-term safety, tolerability, and weight loss associated with metformin in the Diabetes Prevention Program Outcomes Study. Diabetes Care.

[99] Singh S., Bhansali A. (2016). Randomized placebo control study of insulin sensitizers (Metformin and Pioglitazone) in psoriasis patients with metabolic syndrome (Topical Treatment Cohort). BMC Dermatol..

[100] Singh S., Bhansali A. (2017). Randomized placebo control study of metformin in psoriasis patients with metabolic syndrome (systemic treatment cohort). Indian J. Endocrinol. Metab..

[101] Brauchli Y.B., Jick S.S., Curtin F., Meier C.R. (2008). Association between use of thiazolidinediones or other oral antidiabetics and psoriasis: A population based case-control study. J. Am. Acad. Dermatol..

[102] Wu C.Y., Shieh J.J., Shen J.L., Liu Y.Y., Chang Y.T., Chen Y.J. (2015). Association between antidiabetic drugs and psoriasis risk in diabetic patients: Results from a nationwide nested case-control study in Taiwan. J. Am. Acad. Dermatol..

[103] Armstrong A.W., Guerin A., Sundaram M., Wu E.Q., Faust E.S., Ionescu-Ittu R., Mulani P. (2015). Psoriasis and risk of diabetes-associated microvascular and macrovascular complications. J. Am. Acad. Dermatol..

[104] Papagrigoraki A., Del Giglio M., Cosma C., Maurelli M., Girolomoni G., Lapolla A. (2017). Advanced glycation end products are increased in the skin and blood of patients with severe psoriasis. Acta Derm. Venereol..

[105] Dreiher J., Weitzman D., Davidovici B., Shapiro J., Cohen A.D. (2008). Psoriasis and dyslipidaemia: A population-based study. Acta Derm. Venereol..

[106] Miller I.M., Ellervik C., Zarchi K., Ibler K.S., Vinding G.R., Knudsen K.M., Jemec G.B. (2015). The association of metabolic syndrome and psoriasis: A population- and hospital-based cross-sectional study. J. Eur. Acad. Dermatol. Venereol. JEADV.

[107] Ma C., Harskamp C.T., Armstrong E.J., Armstrong A.W. (2013). The association between psoriasis and dyslipidaemia: A systematic review. Br. J. Dermatol..

[108] Santos-Juanes J., Coto-Segura P., Fernandez-Vega I., Armesto S., Martinez-Camblor P. (2015). Psoriasis vulgaris with or without arthritis and independent of disease severity or duration is a risk factor for hypercholesterolemia. Dermatology.

[109] Sunitha S., Rajappa M., Thappa D.M., Chandrashekar L., Munisamy M., Revathy G., Priyadarssini M. (2015). Comprehensive lipid tetrad index, atherogenic index and lipid peroxidation: Surrogate markers for increased cardiovascular risk in psoriasis. Indian J. Dermatol. Venereol. Leprol..

[110] Miller I.M., Skaaby T., Ellervik C., Jemec G.B. (2013). Quantifying cardiovascular disease risk factors in patients with psoriasis: A meta-analysis. Br. J. Dermatol..

[111] Akhyani M., Ehsani A.H., Robati R.M., Robati A.M. (2007). The lipid profile in psoriasis: A controlled study. J. Eur. Acad. Dermatol. Venereol. JEADV.

[112] Reynoso-von Drateln C., Martinez-Abundis E., Balcazar-Munoz B.R., Bustos-Saldana R., Gonzalez-Ortiz M. (2003). Lipid profile, insulin secretion, and insulin sensitivity in psoriasis. J. Am. Acad. Dermatol..

[113] Tom W.L., Playford M.P., Admani S., Natarajan B., Joshi A.A., Eichenfield L.F., Mehta N.N. (2016). Characterization of lipoprotein composition and function in pediatric psoriasis reveals a more atherogenic profile. J. Investig. Dermatol..

[114] Mysliwiec H., Baran A., Harasim-Symbor E., Mysliwiec P., Milewska A.J., Chabowski A., Flisiak I. (2017). Serum fatty acid profile in psoriasis and its comorbidity. Arch. Dermatol. Res..

[115] Wu S., Li W.Q., Han J., Sun Q., Qureshi A.A. (2014). Hypercholesterolemia and risk of incident psoriasis and psoriatic arthritis in US women. Arthritis Rheumatol..

[116] Setty A.R., Curhan G., Choi H.K. (2007). Obesity, waist circumference, weight change, and the risk of psoriasis in women: Nurses’ Health Study II. Arch. Intern. Med..

[117] Correia B., Torres T. (2015). Obesity: A key component of psoriasis. Acta Bio-Med. Atenei Parm..

[118] Duarte G.V., Silva L.P. (2014). Correlation between psoriasis’ severity and waist-to-height ratio. Anais Brasileiros de Dermatologia.

[119] Lee A., Smith S.D., Hong E., Garnett S., Fischer G. (2016). Association between pediatric psoriasis and waist-to-height ratio in the absence of obesity: A multicenter australian study. JAMA Dermatol..

[120] Jacobi A., Langenbruch A., Purwins S., Augustin M., Radtke M.A. (2015). Prevalence of obesity in patients with psoriasis: Results of the national study PsoHealth3. Dermatology.

[121] Fleming P., Kraft J., Gulliver W.P., Lynde C. (2015). The relationship of obesity with the severity of psoriasis: A systematic review. J. Cutan. Med. Surg..

[122] Gutmark-Little I., Shah K.N. (2015). Obesity and the metabolic syndrome in pediatric psoriasis. Clin. Dermatol..

[123] Mahe E., Beauchet A., Bodemer C., Phan A., Bursztejn A.C., Boralevi F., Souillet A.L., Chiaverini C., Bourrat E., Miquel J. (2015). Psoriasis and obesity in French children: A case-control, multicentre study. Br. J. Dermatol..

[124] Torres T., Machado S., Mendonca D., Selores M. (2014). Cardiovascular comorbidities in childhood psoriasis. Eur. J. Dermatol. EJD.

[125] Paller A.S., Mercy K., Kwasny M.J., Choon S.E., Cordoro K.M., Girolomoni G., Menter A., Tom W.L., Mahoney A.M., Oostveen A.M. (2013). Association of pediatric psoriasis severity with excess and central adiposity: An international cross-sectional study. JAMA Dermatol..

[126] Jensen P., Skov L. (2016). Psoriasis and Obesity. Dermatology.

[127] Danielsen K., Wilsgaard T., Olsen A.O., Furberg A.S. (2017). Overweight and weight gain predict psoriasis development in a population-based cohort. Acta Derm. Venereol..

[128] Herron M.D., Hinckley M., Hoffman M.S., Papenfuss J., Hansen C.B., Callis K.P., Krueger G.G. (2005). Impact of obesity and smoking on psoriasis presentation and management. Arch. Dermatol..

[129] Debbaneh M., Millsop J.W., Bhatia B.K., Koo J., Liao W. (2014). Diet and psoriasis, part I: Impact of weight loss interventions. J. Am. Acad. Dermatol..

[130] Jensen P., Christensen R., Zachariae C., Geiker N.R., Schaadt B.K., Stender S., Hansen P.R., Astrup A., Skov L. (2016). Long-term effects of weight reduction on the severity of psoriasis in a cohort derived from a randomized trial: A prospective observational follow-up study. Am. J. Clin. Nutr..

[131] Naldi L., Conti A., Cazzaniga S., Patrizi A., Pazzaglia M., Lanzoni A., Veneziano L., Pellacani G. (2014). Diet and physical exercise in psoriasis: A randomized controlled trial. Br. J. Dermatol..

[132] Roongpisuthipong W., Pongpudpunth M., Roongpisuthipong C., Rajatanavin N. (2013). The effect of weight loss in obese patients with chronic stable plaque-type psoriasis. Dermatol. Res. Pract..

[133] Upala S., Sanguankeo A. (2015). Effect of lifestyle weight loss intervention on disease severity in patients with psoriasis: A systematic review and meta-analysis. Int. J. Obes..

[134] Babino G., Giunta A., Bianchi L., Esposito M. (2017). Morbid obesity and psoriasis: Disease remission after laparoscopic sleeve gastrectomy. Obes. Res. Clin. Pract..

[135] Al-Mutairi N., Nour T. (2014). The effect of weight reduction on treatment outcomes in obese patients with psoriasis on biologic therapy: A randomized controlled prospective trial. Expert Opin. Biol. Ther..

[136] Chiricozzi A., Raimondo A., Lembo S., Fausti F., Dini V., Costanzo A., Monfrecola G., Balato N., Ayala F., Romanelli M. (2016). Crosstalk between skin inflammation and adipose tissue-derived products: Pathogenic evidence linking psoriasis to increased adiposity. Expert Rev. Clin. Immunol..

[137] Coimbra S., Catarino C., Santos-Silva A. (2016). The triad psoriasis-obesity-adipokine profile. J. Eur. Acad. Dermatol. Venereol. JEADV.

[138] Engin A. (2017). The definition and prevalence of obesity and metabolic syndrome. Adv. Exp. Med. Biol..

[139] De la Iglesia R., Loria-Kohen V., Zulet M.A., Martinez J.A., Reglero G., Ramirez de Molina A. (2016). Dietary strategies implicated in the prevention and treatment of metabolic syndrome. Int. J. Mol. Sci..

[140] Danielsen K., Wilsgaard T., Olsen A.O., Eggen A.E., Olsen K., Cassano P.A., Furberg A.S. (2015). Elevated odds of metabolic syndrome in psoriasis: A population-based study of age and sex differences. Br. J. Dermatol..

[141] Milcic D., Jankovic S., Vesic S., Milinkovic M., Marinkovic J., Cirkovic A., Jankovic J. (2017). Prevalence of metabolic syndrome in patients with psoriasis: A hospital-based cross-sectional study. Anais Brasileiros de Dermatologia.

[142] Parodi A., Aste N., Calvieri C., Cantoresi F., Carlesimo M., Fabbri P., Filosa G., Galluccio A., Lisi P., Micali G. (2014). Metabolic syndrome prevalence in psoriasis: A cross-sectional study in the Italian population. Am. J. Clin. Dermatol..

[143] Rodriguez-Zuniga M.J.M., Cortez-Franco F., Quijano-Gomero E. (2017). Association of psoriasis and metabolic syndrome in Latin America: A systematic review and meta-analysis. Actas Derm. Sifiliogr..

[144] Malkic Salihbegovic E., Hadzigrahic N., Cickusic A.J. (2015). Psoriasis and metabolic syndrome. Med. Arch..

[145] Singh S., Young P., Armstrong A.W. (2016). Relationship between psoriasis and metabolic syndrome: A systematic review. Giornale Italiano di Dermatologia e Venereologia.

[146] Kokpol C., Aekplakorn W., Rajatanavin N. (2014). Prevalence and characteristics of metabolic syndrome in South-East Asian psoriatic patients: A case-control study. J. Dermatol..

[147] Gui X.Y., Yu X.L., Jin H.Z., Zuo Y.G., Wu C. (2017). Prevalence of metabolic syndrome in Chinese psoriasis patients: A hospital-based cross-sectional study. J. Diabetes Investig..

[148] Sharma Y.K., Prakash N., Gupta A. (2016). Prevalence of metabolic syndrome as per the NCEP and IDF definitions vis-a-vis severity and duration of psoriasis in a semi-urban Maharashtrian population: A case control study. Diabetes Metab. Syndr..

[149] Pietrzak A., Grywalska E., Walankiewicz M., Lotti T., Rolinski J., Myslinski W., Chabros P., Piekarska-Myslinska D., Reich K. (2017). Psoriasis and metabolic syndrome in children: Current data. Clin. Exp. Dermatol..

[150] Lonnberg A.S., Skov L., Skytthe A., Kyvik K.O., Pedersen O.B., Thomsen S.F. (2016). Smoking and risk for psoriasis: A population-based twin study. Int. J. Dermatol..

[151] Adamzik K., McAleer M.A., Kirby B. (2013). Alcohol and psoriasis: Sobering thoughts. Clin. Exp. Dermatol..

[152] Armstrong A.W., Harskamp C.T., Dhillon J.S., Armstrong E.J. (2014). Psoriasis and smoking: A systematic review and meta-analysis. Br. J. Dermatol..

[153] Brenaut E., Horreau C., Pouplard C., Barnetche T., Paul C., Richard M.A., Joly P., Le Maitre M., Aractingi S., Aubin F. (2013). Alcohol consumption and psoriasis: A systematic literature review. J. Eur. Acad. Dermatol. Venereol. JEADV.

[154] Li W., Han J., Choi H.K., Qureshi A.A. (2012). Smoking and risk of incident psoriasis among women and men in the United States: A combined analysis. Am. J. Epidemiol..

[155] Setty A.R., Curhan G., Choi H.K. (2007). Smoking and the risk of psoriasis in women: Nurses’ Health Study II. Am. J. Med..

[156] Naldi L., Peli L., Parazzini F. (1999). Association of early-stage psoriasis with smoking and male alcohol consumption: Evidence from an Italian case-control study. Arch. Dermatol..

[157] Fortes C., Mastroeni S., Leffondre K., Sampogna F., Melchi F., Mazzotti E., Pasquini P., Abeni D. (2005). Relationship between smoking and the clinical severity of psoriasis. Arch. Dermatol..

[158] Richer V., Roubille C., Fleming P., Starnino T., McCourt C., McFarlane A., Siu S., Kraft J., Lynde C., Pope J.E. (2016). Psoriasis and Smoking: A systematic literature review and meta-analysis with qualitative analysis of effect of smoking on psoriasis severity. J. Cutan. Med. Surg..

[159] Torii K., Saito C., Furuhashi T., Nishioka A., Shintani Y., Kawashima K., Kato H., Morita A. (2011). Tobacco smoke is related to Th17 generation with clinical implications for psoriasis patients. Exp. Dermatol..

[160] Ku S.H., Kwon W.J., Cho E.B., Park E.J., Kim K.H., Kim K.J. (2016). The association between psoriasis area and severity index and cardiovascular risk factor in korean psoriasis patients. Ann. Dermatol..

[161] Irimie M., Oanta A., Irimie C.A., Fekete L.G., Minea D.I., Pascu A. (2015). Cardiovascular risk factors in patients with chronic plaque psoriasis: A case-control study on the Brasov County population. Acta Dermatovenerol. Croat. ADC.

[162] Al-Mutairi N., Al-Farag S., Al-Mutairi A., Al-Shiltawy M. (2010). Comorbidities associated with psoriasis: An experience from the Middle East. J. Dermatol..

[163] Armstrong A.W., Schupp C., Bebo B. (2012). Psoriasis comorbidities: Results from the National Psoriasis Foundation surveys 2003 to 2011. Dermatology.

[164] Curco N., Barriendos N., Barahona M.J., Arteaga C., Garcia M., Yordanov S., de La Barrera O., Prat C., Vives P., Gimenez N. (2017). Factors influencing cardiometabolic risk profile in patients with psoriasis. Australas. J. Dermatol..

[165] Itani S., Arabi A., Harb D., Hamzeh D., Kibbi A.G. (2016). High prevalence of metabolic syndrome in patients with psoriasis in Lebanon: A prospective study. Int. J. Dermatol..

[166] Hagforsen E., Michaelsson K., Lundgren E., Olofsson H., Petersson A., Lagumdzija A., Hedstrand H., Michaelsson G. (2005). Women with palmoplantar pustulosis have disturbed calcium homeostasis and a high prevalence of diabetes mellitus and psychiatric disorders: A case-control study. Acta Derm. Venereol..

[167] Zhang C., Zhu K., Zhou H., Liu J., Xu G., Liu W. (2015). Metabolic abnormalities are absent in patients with generalized pustular psoriasis. J. Dermatol. Sci..

[168] Eder L., Wu Y., Chandran V., Cook R., Gladman D.D. (2016). Incidence and predictors for cardiovascular events in patients with psoriatic arthritis. Ann. Rheum. Dis..

[169] Li L., Hagberg K.W., Peng M., Shah K., Paris M., Jick S. (2015). Rates of cardiovascular disease and major adverse cardiovascular events in patients with psoriatic arthritis compared to patients without psoriatic arthritis. J. Clin. Rheumatol..

[170] Polachek A., Touma Z., Anderson M., Eder L. (2017). Risk of Cardiovascular morbidity in patients with psoriatic arthritis: A meta-analysis of observational studies. Arthritis Care Res..

[171] Horreau C., Pouplard C., Brenaut E., Barnetche T., Misery L., Cribier B., Jullien D., Aractingi S., Aubin F., Joly P. (2013). Cardiovascular morbidity and mortality in psoriasis and psoriatic arthritis: A systematic literature review. J. Eur. Acad. Dermatol. Venereol. JEADV.

[172] Di Minno M.N., Ambrosino P., Lupoli R., di Minno A., Tasso M., Peluso R., Tremoli E. (2015). Cardiovascular risk markers in patients with psoriatic arthritis: A meta-analysis of literature studies. Ann. Med..

[173] Shen J., Wong K.T., Cheng I.T., Shang Q., Li E.K., Wong P., Kun E.W., Law M.Y., Yip R., Yim I. (2017). Increased prevalence of coronary plaque in patients with psoriatic arthritis without prior diagnosis of coronary artery disease. Ann. Rheum. Dis..

[174] Eder L., Abji F., Rosen C.F., Chandran V., Gladman D.D. (2017). The association between obesity and clinical features of psoriatic arthritis: A case-control study. J. Rheumatol..

[175] Eder L., Chandran V., Cook R., Gladman D.D. (2017). The risk of developing diabetes mellitus in patients with psoriatic arthritis: A cohort study. J. Rheumatol..

[176] Gulati A.M., Semb A.G., Rollefstad S., Romundstad P.R., Kavanaugh A., Gulati S., Haugeberg G., Hoff M. (2016). On the HUNT for cardiovascular risk factors and disease in patients with psoriatic arthritis: Population-based data from the Nord-Trondelag Health Study. Ann. Rheum. Dis..

[177] Haroon M., Gallagher P., Heffernan E., FitzGerald O. (2014). High prevalence of metabolic syndrome and of insulin resistance in psoriatic arthritis is associated with the severity of underlying disease. J. Rheumatol..

[178] Jafri K., Bartels C.M., Shin D., Gelfand J.M., Ogdie A. (2017). Incidence and management of cardiovascular risk factors in psoriatic arthritis and rheumatoid arthritis: A population-based study. Arthritis Care Res..

[179] Chin Y.Y., Yu H.S., Li W.C., Ko Y.C., Chen G.S., Wu C.S., Lu Y.W., Yang Y.H., Lan C.C. (2013). Arthritis as an important determinant for psoriatic patients to develop severe vascular events in Taiwan: A nation-wide study. J. Eur. Acad. Dermatol. Venereol. JEADV.

[180] Lu Y., Chen H., Nikamo P., Qi Low H., Helms C., Seielstad M., Liu J., Bowcock A.M., Stahle M., Liao W. (2013). Association of cardiovascular and metabolic disease genes with psoriasis. J. Investig. Dermatol..

[181] Wolf N., Quaranta M., Prescott N.J., Allen M., Smith R., Burden A.D., Worthington J., Griffiths C.E., Mathew C.G., Barker J.N. (2008). Psoriasis is associated with pleiotropic susceptibility loci identified in type II diabetes and Crohn disease. J. Med. Genet..

[182] Campalani E., Allen M.H., Fairhurst D., Young H.S., Mendonca C.O., Burden A.D., Griffiths C.E., Crook M.A., Barker J.N., Smith C.H. (2006). Apolipoprotein E gene polymorphisms are associated with psoriasis but do not determine disease response to acitretin. Br. J. Dermatol..

[183] Eiris N., Gonzalez-Lara L., Santos-Juanes J., Queiro R., Coto E., Coto-Segura P. (2014). Genetic variation at IL12B, IL23R and IL23A is associated with psoriasis severity, psoriatic arthritis and type 2 diabetes mellitus. J. Dermatol. Sci..

[184] Koch M., Baurecht H., Ried J.S., Rodriguez E., Schlesinger S., Volks N., Gieger C., Ruckert I.M., Heinrich L., Willenborg C. (2015). Psoriasis and cardiometabolic traits: Modest association but distinct genetic architectures. J. Investig. Dermatol..

[185] Gupta Y., Moller S., Zillikens D., Boehncke W.H., Ibrahim S.M., Ludwig R.J. (2013). Genetic control of psoriasis is relatively distinct from that of metabolic syndrome and coronary artery disease. Exp. Dermatol..

[186] Davidovici B.B., Sattar N., Prinz J., Puig L., Emery P., Barker J.N., van de Kerkhof P., Stahle M., Nestle F.O., Girolomoni G. (2010). Psoriasis and systemic inflammatory diseases: Potential mechanistic links between skin disease and co-morbid conditions. J. Investig. Dermatol..

[187] Spah F. (2008). Inflammation in atherosclerosis and psoriasis: Common pathogenic mechanisms and the potential for an integrated treatment approach. Br. J. Dermatol..

[188] Hjuler K.F., Gormsen L.C., Vendelbo M.H., Egeberg A., Nielsen J., Iversen L. (2017). Increased global arterial and subcutaneous adipose tissue inflammation in patients with moderate-to-severe psoriasis. Br. J. Dermatol..

[189] Rose S., Sheth N.H., Baker J.F., Ogdie A., Raper A., Saboury B., Werner T.J., Thomas P., Vanvoorhees A., Alavi A. (2013). A comparison of vascular inflammation in psoriasis, rheumatoid arthritis, and healthy subjects by FDG-PET/CT: A pilot study. Am. J. Cardiovasc. Dis..

[190] Youn S.W., Kang S.Y., Kim S.A., Park G.Y., Lee W.W. (2015). Subclinical systemic and vascular inflammation detected by (18) F-fluorodeoxyglucose positron emission tomography/computed tomography in patients with mild psoriasis. J. Dermatol..

[191] Mehta N.N., Yu Y., Saboury B., Foroughi N., Krishnamoorthy P., Raper A., Baer A., Antigua J., Van Voorhees A.S., Torigian D.A. (2011). Systemic and vascular inflammation in patients with moderate to severe psoriasis as measured by [18F]-fluorodeoxyglucose positron emission tomography-computed tomography (FDG-PET/CT): A pilot study. Arch. Dermatol..

[192] Wang Y., Gao H., Loyd C.M., Fu W., Diaconu D., Liu S., Cooper K.D., McCormick T.S., Simon D.I., Ward N.L. (2012). Chronic skin-specific inflammation promotes vascular inflammation and thrombosis. J. Investig. Dermatol..

[193] Chodorowska G., Wojnowska D., Juszkiewicz-Borowiec M. (2004). C-reactive protein and alpha2-macroglobulin plasma activity in medium-severe and severe psoriasis. J. Eur. Acad. Dermatol. Venereol. JEADV.

[194] Coimbra S., Oliveira H., Reis F., Belo L., Rocha S., Quintanilha A., Figueiredo A., Teixeira F., Castro E., Rocha-Pereira P. (2010). C-reactive protein and leucocyte activation in psoriasis vulgaris according to severity and therapy. J. Eur. Acad. Dermatol. Venereol. JEADV.

[195] Tian S., Krueger J.G., Li K., Jabbari A., Brodmerkel C., Lowes M.A., Suarez-Farinas M. (2012). Meta-analysis derived (MAD) transcriptome of psoriasis defines the “core” pathogenesis of disease. PLoS ONE.

[196] Boehncke W.H., Boehncke S., Tobin A.M., Kirby B. (2011). The ‘psoriatic march’: A concept of how severe psoriasis may drive cardiovascular comorbidity. Exp. Dermatol..

[197] Armstrong A.W., Voyles S.V., Armstrong E.J., Fuller E.N., Rutledge J.C. (2011). A tale of two plaques: Convergent mechanisms of T-cell-mediated inflammation in psoriasis and atherosclerosis. Exp. Dermatol..

[198] Grozdev I., Korman N., Tsankov N. (2014). Psoriasis as a systemic disease. Clin. Dermatol..

[199] Butcher M., Galkina E. (2011). Current views on the functions of interleukin-17A-producing cells in atherosclerosis. Thromb. Haemost..

[200] Furuhashi T., Saito C., Torii K., Nishida E., Yamazaki S., Morita A. (2013). Photo(chemo)therapy reduces circulating Th17 cells and restores circulating regulatory T cells in psoriasis. PLoS ONE.

[201] Lo Y.H., Torii K., Saito C., Furuhashi T., Maeda A., Morita A. (2010). Serum IL-22 correlates with psoriatic severity and serum IL-6 correlates with susceptibility to phototherapy. J. Dermatol. Sci..

[202] Qu Y., Zhang Q., Ma S., Liu S., Chen Z., Mo Z., You Z. (2016). Interleukin-17A differentially induces inflammatory and metabolic gene expression in the adipose tissues of lean and obese mice. Int. J. Mol. Sci..

[203] Hashmi S., Zeng Q.T. (2006). Role of interleukin-17 and interleukin-17-induced cytokines interleukin-6 and interleukin-8 in unstable coronary artery disease. Coron. Artery Dis..

[204] Karbach S., Croxford A.L., Oelze M., Schuler R., Minwegen D., Wegner J., Koukes L., Yogev N., Nikolaev A., Reissig S. (2014). Interleukin 17 drives vascular inflammation, endothelial dysfunction, and arterial hypertension in psoriasis-like skin disease. Arterioscler. Thromb. Vasc. Biol..

[205] Owczarczyk-Saczonek A., Placek W. (2017). Interleukin-17 as a factor linking the pathogenesis of psoriasis with metabolic disorders. Int. J. Dermatol..

[206] Mehta N.N., Li K., Szapary P., Krueger J., Brodmerkel C. (2013). Modulation of cardiometabolic pathways in skin and serum from patients with psoriasis. J. Trans. Med..

[207] Furue M., Kadono T. (2017). “Inflammatory skin march” in atopic dermatitis and psoriasis. Inflamm. Res..

[208] Deng Y., Scherer P.E. (2010). Adipokines as novel biomarkers and regulators of the metabolic syndrome. Ann. N.Y. Acad. Sci..

[209] Chen Y.J., Wu C.Y., Shen J.L., Chu S.Y., Chen C.K., Chang Y.T., Chen C.M. (2008). Psoriasis independently associated with hyperleptinemia contributing to metabolic syndrome. Arch. Dermatol..

[210] Cerman A.A., Bozkurt S., Sav A., Tulunay A., Elbasi M.O., Ergun T. (2008). Serum leptin levels, skin leptin and leptin receptor expression in psoriasis. Br. J. Dermatol..

[211] Oh Y.J., Lim H.K., Choi J.H., Lee J.W., Kim N.I. (2014). Serum leptin and adiponectin levels in Korean patients with psoriasis. J. Korean Med. Sci..

[212] Stjernholm T., Ommen P., Langkilde A., Johansen C., Iversen L., Rosada C., Stenderup K. (2017). Leptin deficiency in mice counteracts imiquimod (IMQ)-induced psoriasis-like skin inflammation while leptin stimulation induces inflammation in human keratinocytes. Exp. Dermatol..

[213] Pina T., Genre F., Lopez-Mejias R., Armesto S., Ubilla B., Mijares V., Dierssen-Sotos T., Gonzalez-Lopez M.A., Gonzalez-Vela M.C., Blanco R. (2015). Relationship of leptin with adiposity and inflammation and resistin with disease severity in psoriatic patients undergoing anti-TNF-alpha therapy. J. Eur. Acad. Dermatol. Venereol. JEADV.

[214] Robati R.M., Partovi-Kia M., Haghighatkhah H.R., Younespour S., Abdollahimajd F. (2014). Increased serum leptin and resistin levels and increased carotid intima-media wall thickness in patients with psoriasis: Is psoriasis associated with atherosclerosis?. J. Am. Acad. Dermatol..

[215] Li R.C., Krishnamoorthy P., DerOhannessian S., Doveikis J., Wilcox M., Thomas P., Rader D.J., Reilly M.P., Van Voorhees A., Gelfand J.M. (2014). Psoriasis is associated with decreased plasma adiponectin levels independently of cardiometabolic risk factors. Clin. Exp. Dermatol..

[216] Johnston A., Arnadottir S., Gudjonsson J.E., Aphale A., Sigmarsdottir A.A., Gunnarsson S.I., Steinsson J.T., Elder J.T., Valdimarsson H. (2008). Obesity in psoriasis: Leptin and resistin as mediators of cutaneous inflammation. Br. J. Dermatol..

[217] Okan G., Baki A.M., Yorulmaz E., Dogru-Abbasoglu S., Vural P. (2016). Serum visfatin, fetuin-A, and pentraxin 3 levels in patients with psoriasis and their relation to disease severity. J. Clin. Lab. Anal..

[218] He L., Qin S., Dang L., Song G., Yao S., Yang N., Li Y. (2014). Psoriasis decreases the anti-oxidation and anti-inflammation properties of high-density lipoprotein. Biochim. Biophys. Acta.

[219] Holzer M., Wolf P., Inzinger M., Trieb M., Curcic S., Pasterk L., Weger W., Heinemann A., Marsche G. (2014). Anti-psoriatic therapy recovers high-density lipoprotein composition and function. J. Investig. Dermatol..

[220] Chua R.A., Arbiser J.L. (2009). The role of angiogenesis in the pathogenesis of psoriasis. Autoimmunity.

[221] Sluimer J.C., Daemen M.J. (2009). Novel concepts in atherogenesis: Angiogenesis and hypoxia in atherosclerosis. J. Pathol..

[222] Xu J., Lu X., Shi G.P. (2015). Vasa vasorum in atherosclerosis and clinical significance. Int. J. Mol. Sci..

[223] Armstrong A.W., Voyles S.V., Armstrong E.J., Fuller E.N., Rutledge J.C. (2011). Angiogenesis and oxidative stress: Common mechanisms linking psoriasis with atherosclerosis. J. Dermatol. Sci..

[224] Malecic N., Young H.S. (2017). Excessive angiogenesis associated with psoriasis as a cause for cardiovascular ischaemia. Exp. Dermatol..

[225] Rocha-Pereira P., Santos-Silva A., Rebelo I., Figueiredo A., Quintanilha A., Teixeira F. (2001). Dislipidemia and oxidative stress in mild and in severe psoriasis as a risk for cardiovascular disease. Clin. Chim. Acta Int. J. Clin. Chem..

[226] He F., Zuo L. (2015). Redox roles of reactive oxygen species in cardiovascular diseases. Int. J. Mol. Sci..

[227] Baron M., Boulanger C.M., Staels B., Tailleux A. (2012). Cell-derived microparticles in atherosclerosis: Biomarkers and targets for pharmacological modulation?. J. Cell. Mol. Med..

[228] Takeshita J., Mohler E.R., Krishnamoorthy P., Moore J., Rogers W.T., Zhang L., Gelfand J.M., Mehta N.N. (2014). Endothelial cell-, platelet-, and monocyte/macrophage-derived microparticles are elevated in psoriasis beyond cardiometabolic risk factors. J. Am. Heart Assoc..

[229] Tamagawa-Mineoka R., Katoh N., Kishimoto S. (2010). Platelet activation in patients with psoriasis: Increased plasma levels of platelet-derived microparticles and soluble P-selectin. J. Am. Acad. Dermatol..

[230] Papadavid E., Diamanti K., Spathis A., Varoudi M., Andreadou I., Gravanis K., Theodoropoulos K., Karakitsos P., Lekakis J., Rigopoulos D. (2016). Increased levels of circulating platelet-derived microparticles in psoriasis: Possible implications for the associated cardiovascular risk. World J. Cardiol..

[231] Kasperska-Zajac A., Brzoza Z., Rogala B. (2008). Platelet function in cutaneous diseases. Platelets.

[232] Chandrashekar L., Rajappa M., Revathy G., Sundar I., Munisamy M., Ananthanarayanan P.H., Thappa D.M., Basu D. (2015). Is enhanced platelet activation the missing link leading to increased cardiovascular risk in psoriasis?. Clin. Chim. Acta Int. J. Clin. Chem..

[233] Nielsen H.J., Christensen I.J., Svendsen M.N., Hansen U., Werther K., Brunner N., Petersen L.J., Kristensen J.K. (2002). Elevated plasma levels of vascular endothelial growth factor and plasminogen activator inhibitor-1 decrease during improvement of psoriasis. Inflamm. Res..

[234] Graham I.M., Daly L.E., Refsum H.M., Robinson K., Brattstrom L.E., Ueland P.M., Palma-Reis R.J., Boers G.H., Sheahan R.G., Israelsson B. (1997). Plasma homocysteine as a risk factor for vascular disease. The European Concerted Action Project. JAMA.

[235] Skovierova H., Vidomanova E., Mahmood S., Sopkova J., Drgova A., Cervenova T., Halasova E., Lehotsky J. (2016). The molecular and cellular effect of homocysteine metabolism imbalance on human health. Int. J. Mol. Sci..

[236] Malerba M., Gisondi P., Radaeli A., Sala R., Calzavara Pinton P.G., Girolomoni G. (2006). Plasma homocysteine and folate levels in patients with chronic plaque psoriasis. Br. J. Dermatol..

[237] Gerdes S., Osadtschy S., Buhles N., Baurecht H., Mrowietz U. (2014). Cardiovascular biomarkers in patients with psoriasis. Exp. Dermatol..

[238] Takahashi H., Iinuma S., Honma M., Iizuka H. (2014). Increased serum C-reactive protein level in Japanese patients of psoriasis with cardio- and cerebrovascular disease. J. Dermatol..

[239] Erturan I., Koroglu B.K., Adiloglu A., Ceyhan A.M., Akkaya V.B., Tamer N., Basak P.Y., Korkmaz S., Ersoy I.H., Kilinc O. (2014). Evaluation of serum sCD40L and homocysteine levels with subclinical atherosclerosis indicators in patients with psoriasis: A pilot study. Int. J. Dermatol..

[240] Baran A., Mysliwiec H., Szterling-Jaworowska M., Kiluk P., Swiderska M., Flisiak I. (2017). Serum YKL-40 as a potential biomarker of inflammation in psoriasis. J. Dermatol. Treat..

[241] Joshi A.A., Lerman J.B., Aberra T.M., Afshar M., Teague H.L., Rodante J.A., Krishnamoorthy P., Ng Q., Aridi T.Z., Salahuddin T. (2016). GlycA is a novel biomarker of inflammation and subclinical cardiovascular disease in psoriasis. Circ. Res..

[242] Torres T., Bettencourt N., Mendonca D., Vasconcelos C., Silva B.M., Selores M. (2014). Complement C3 as a marker of cardiometabolic risk in psoriasis. Arch. Dermatol. Res..

[243] Yurtdas M., Yaylali Y.T., Kaya Y., Ozdemir M., Ozkan I., Aladag N. (2014). Neutrophil-to-lymphocyte ratio may predict subclinical atherosclerosis in patients with psoriasis. Echocardiography.

[244] Kim D.S., Shin D., Lee M.S., Kim H.J., Kim D.Y., Kim S.M., Lee M.G. (2016). Assessments of neutrophil to lymphocyte ratio and platelet to lymphocyte ratio in Korean patients with psoriasis vulgaris and psoriatic arthritis. J. Dermatol..

[245] Cao L.Y., Soler D.C., Debanne S.M., Grozdev I., Rodriguez M.E., Feig R.L., Carman T.L., Gilkeson R.C., Orringer C.E., Kern E.F. (2013). Psoriasis and cardiovascular risk factors: Increased serum myeloperoxidase and corresponding immunocellular overexpression by Cd11b(+) CD68(+) macrophages in skin lesions. Am. J. Transl. Res..

[246] Menter A., Korman N.J., Elmets C.A., Feldman S.R., Gelfand J.M., Gordon K.B., Gottlieb A.B., Koo J.Y., Lebwohl M., Lim H.W. (2009). Guidelines of care for the management of psoriasis and psoriatic arthritis: Section 4. Guidelines of care for the management and treatment of psoriasis with traditional systemic agents. J. Am. Acad. Dermatol..

[247] Mehta D., Lim H.W. (2016). Ultraviolet B phototherapy for psoriasis: review of practical guidelines. Am. J. Clin. Dermatol..

[248] Hugh J., Van Voorhees A.S., Nijhawan R.I., Bagel J., Lebwohl M., Blauvelt A., Hsu S., Weinberg J.M. (2014). From the Medical Board Of The National Psoriasis Foundation: The risk of cardiovascular disease in individuals with psoriasis and the potential impact of current therapies. J. Am. Acad. Dermatol..

[249] Sigurdardottir G., Ekman A.K., Stahle M., Bivik C., Enerback C. (2014). Systemic treatment and narrowband ultraviolet B differentially affect cardiovascular risk markers in psoriasis. J. Am. Acad. Dermatol..

[250] Churton S., Brown L., Shin T.M., Korman N.J. (2014). Does treatment of psoriasis reduce the risk of cardiovascular disease?. Drugs.

[251] Prodanovich S., Ma F., Taylor J.R., Pezon C., Fasihi T., Kirsner R.S. (2005). Methotrexate reduces incidence of vascular diseases in veterans with psoriasis or rheumatoid arthritis. J. Am. Acad. Dermatol..

[252] Ahlehoff O., Skov L., Gislason G., Gniadecki R., Iversen L., Bryld L.E., Lasthein S., Lindhardsen J., Kristensen S.L., Torp-Pedersen C. (2015). Cardiovascular outcomes and systemic anti-inflammatory drugs in patients with severe psoriasis: 5-year follow-up of a Danish nationwide cohort. J. Eur. Acad. Dermatol. Venereol. JEADV.

[253] Gulliver W.P., Young H.M., Bachelez H., Randell S., Gulliver S., Al-Mutairi N. (2016). Psoriasis patients treated with biologics and methotrexate have a reduced rate of myocardial infarction: A collaborative analysis using international cohorts. J. Cutan. Med. Surg..

[254] Ahlehoff O., Skov L., Gislason G., Lindhardsen J., Kristensen S.L., Iversen L., Lasthein S., Gniadecki R., Dam T.N., Torp-Pedersen C. (2013). Cardiovascular disease event rates in patients with severe psoriasis treated with systemic anti-inflammatory drugs: A Danish real-world cohort study. J. Intern. Med..

[255] Mahmoudi M.J., Saboor-Yaraghi A.A., Zabetian-Targhi F., Siassi F., Zarnani A.H., Eshraghian M.R., Shokri F., Rezaei N., Kalikias Y., Mahmoudi M. (2016). Vitamin A decreases cytotoxicity of oxidized low-density lipoprotein in patients with atherosclerosis. Immunol. Investig..

[256] Zhou W., Lin J., Chen H., Wang J., Liu Y., Xia M. (2015). Retinoic acid induces macrophage cholesterol efflux and inhibits atherosclerotic plaque formation in apoE-deficient mice. Br. J. Nutr..

[257] Relevy N.Z., Harats D., Harari A., Ben-Amotz A., Bitzur R., Ruhl R., Shaish A. (2015). Vitamin A-deficient diet accelerated atherogenesis in apolipoprotein E(-/-) mice and dietary beta-carotene prevents this consequence. BioMed Res. Int..

[258] Mottaghi A., Ebrahimof S., Angoorani P., Saboor-Yaraghi A.A. (2014). Vitamin A supplementation reduces IL-17 and RORc gene expression in atherosclerotic patients. Scand. J. Immunol..

[259] Bilbija D., Elmabsout A.A., Sagave J., Haugen F., Bastani N., Dahl C.P., Gullestad L., Sirsjo A., Blomhoff R., Valen G. (2014). Expression of retinoic acid target genes in coronary artery disease. Int. J. Mol. Med..

[260] Mottaghi A., Salehi E., Keshvarz A., Sezavar H., Saboor-Yaraghi A.A. (2012). The influence of vitamin A supplementation on Foxp3 and TGF-beta gene expression in atherosclerotic patients. J. Nutrigenet. Nutrigenom..

[261] Zhou B., Pan Y., Hu Z., Wang X., Han J., Zhou Q., Zhai Z., Wang Y. (2012). All-trans-retinoic acid ameliorated high fat diet-induced atherosclerosis in rabbits by inhibiting platelet activation and inflammation. J. Biomed. Biotechnol..

[262] Abuabara K., Lee H., Kimball A.B. (2011). The effect of systemic psoriasis therapies on the incidence of myocardial infarction: A cohort study. Br. J. Dermatol..

[263] Wu J.J., Poon K.Y., Channual J.C., Shen A.Y. (2012). Association between tumor necrosis factor inhibitor therapy and myocardial infarction risk in patients with psoriasis. Arch. Dermatol..

[264] Wu J.J., Poon K.Y. (2013). Association of ethnicity, tumor necrosis factor inhibitor therapy, and myocardial infarction risk in patients with psoriasis. J. Am. Acad. Dermatol..

[265] Wu J.J., Poon K.Y., Bebchuk J.D. (2013). Association between the type and length of tumor necrosis factor inhibitor therapy and myocardial infarction risk in patients with psoriasis. J. Drugs Dermatol. JDD.

[266] Wu J.J., Poon K.Y., Bebchuk J.D. (2014). Tumor necrosis factor inhibitor therapy and myocardial infarction risk in patients with psoriasis, psoriatic arthritis, or both. J. Drugs Dermatol. JDD.

[267] Wu J.J., Guerin A., Sundaram M., Dea K., Cloutier M., Mulani P. (2017). Cardiovascular event risk assessment in psoriasis patients treated with tumor necrosis factor-alpha inhibitors versus methotrexate. J. Am. Acad. Dermatol..

[268] Boehncke S., Fichtlscherer S., Salgo R., Garbaraviciene J., Beschmann H., Diehl S., Hardt K., Thaci D., Boehncke W.H. (2011). Systemic therapy of plaque-type psoriasis ameliorates endothelial cell function: Results of a prospective longitudinal pilot trial. Arch. Dermatol. Res..

[269] Schmieder A., Poppe M., Hametner C., Meyer-Schraml H., Schaarschmidt M.L., Findeisen P., Benoit S., Bauer B., Schmid S., Goebeler M. (2015). Impact of fumaric acid esters on cardiovascular risk factors and depression in psoriasis: A prospective pilot study. Arch. Dermatol. Res..

[270] Famenini S., Sako E.Y., Wu J.J. (2014). Effect of treating psoriasis on cardiovascular co-morbidities: Focus on TNF inhibitors. Am. J. Clin. Dermatol..

[271] Nguyen T., Wu J.J. (2014). Relationship between tumor necrosis factor-alpha inhibitors and cardiovascular disease in psoriasis: A review. Perm. J..

[272] Shaaban D., Al-Mutairi N. (2016). The effect of tumour necrosis factor inhibitor therapy on the incidence of myocardial infarction in patients with psoriasis: A retrospective study. J. Dermatol. Treat..

[273] Yang Z.S., Lin N.N., Li L., Li Y. (2016). The effect of TNF inhibitors on cardiovascular events in psoriasis and psoriatic arthritis: An updated meta-analysis. Clin. Rev. Allergy Immunol..

[274] Piaserico S., Osto E., Famoso G., Zanetti I., Gregori D., Poretto A., Iliceto S., Peserico A., Tona F. (2016). Treatment with tumor necrosis factor inhibitors restores coronary microvascular function in young patients with severe psoriasis. Atherosclerosis.

[275] Hjuler K.F., Bottcher M., Vestergaard C., Botker H.E., Iversen L., Kragballe K. (2016). Association between changes in coronary artery disease progression and treatment with biologic agents for severe psoriasis. JAMA Dermatol..

[276] Pina T., Corrales A., Lopez-Mejias R., Armesto S., Gonzalez-Lopez M.A., Gomez-Acebo I., Ubilla B., Remuzgo-Martinez S., Gonzalez-Vela M.C., Blanco R. (2016). Anti-tumor necrosis factor-alpha therapy improves endothelial function and arterial stiffness in patients with moderate to severe psoriasis: A 6-month prospective study. J. Dermatol..

[277] Bissonnette R., Harel F., Krueger J.G., Guertin M.C., Chabot-Blanchet M., Gonzalez J., Maari C., Delorme I., Lynde C.W., Tardif J.C. (2017). TNF-alpha antagonist and vascular inflammation in patients with psoriasis vulgaris: A randomized placebo-controlled study. J. Investig. Dermatol..

[278] Solomon D.H., Massarotti E., Garg R., Liu J., Canning C., Schneeweiss S. (2011). Association between disease-modifying antirheumatic drugs and diabetes risk in patients with rheumatoid arthritis and psoriasis. JAMA.

[279] Al-Mutairi N., Shabaan D. (2016). Effects of tumor necrosis factor alpha inhibitors extend beyond psoriasis: Insulin sensitivity in psoriasis patients with type 2 diabetes mellitus. Cutis.

[280] Pina T., Armesto S., Lopez-Mejias R., Genre F., Ubilla B., Gonzalez-Lopez M.A., Gonzalez-Vela M.C., Corrales A., Blanco R., Garcia-Unzueta M.T. (2015). Anti-TNF-alpha therapy improves insulin sensitivity in non-diabetic patients with psoriasis: A 6-month prospective study. J. Eur. Acad. Dermatol. Venereol. JEADV.

[281] Wu J.J., Rowan C.G., Bebchuk J.D., Anthony M.S. (2015). No association between TNF inhibitor and methotrexate therapy versus methotrexate in changes in hemoglobin A1C and fasting glucose among psoriasis, psoriatic arthritis, and rheumatoid arthritis patients. J. Drugs Dermatol. JDD.

[282] Gisondi P., Cotena C., Tessari G., Girolomoni G. (2008). Anti-tumour necrosis factor-alpha therapy increases body weight in patients with chronic plaque psoriasis: A retrospective cohort study. J. Eur. Acad. Dermatol. Venereol. JEADV.

[283] Renzo L.D., Saraceno R., Schipani C., Rizzo M., Bianchi A., Noce A., Esposito M., Tiberti S., Chimenti S., A D.E.L. (2011). Prospective assessment of body weight and body composition changes in patients with psoriasis receiving anti-TNF-alpha treatment. Dermatol. Ther..

[284] Saraceno R., Schipani C., Mazzotta A., Esposito M., di Renzo L., de Lorenzo A., Chimenti S. (2008). Effect of anti-tumor necrosis factor-alpha therapies on body mass index in patients with psoriasis. Pharmacol. Res..

[285] Gkalpakiotis S., Arenbergerova M., Gkalpakioti P., Potockova J., Arenberger P., Kraml P. (2017). Impact of adalimumab treatment on cardiovascular risk biomarkers in psoriasis: Results of a pilot study. J. Dermatol..

[286] Genre F., Armesto S., Corrales A., Lopez-Mejias R., Remuzgo-Martinez S., Pina T., Ubilla B., Mijares V., Martin-Varillas J.L., Rueda-Gotor J. (2017). Significant sE-Selectin levels reduction after 6 months of anti-TNF-alpha therapy in non-diabetic patients with moderate-to-severe psoriasis. J. Dermatol. Treat..

[287] Strober B., Teller C., Yamauchi P., Miller J.L., Hooper M., Yang Y.C., Dann F. (2008). Effects of etanercept on C-reactive protein levels in psoriasis and psoriatic arthritis. Br. J. Dermatol..

[288] Kanelleas A., Liapi C., Katoulis A., Stavropoulos P., Avgerinou G., Georgala S., Economopoulos T., Stavrianeas N.G., Katsambas A. (2011). The role of inflammatory markers in assessing disease severity and response to treatment in patients with psoriasis treated with etanercept. Clin. Exp. Dermatol..

[289] Campanati A., Ganzetti G., Giuliodori K., Marra M., Bonfigli A., Testa R., Offidani A. (2015). Serum levels of adipocytokines in psoriasis patients receiving tumor necrosis factor-alpha inhibitors: Results of a retrospective analysis. Int. J. Dermatol..

[290] Pelletier F., Garnache-Ottou F., Biichle S., Vivot A., Humbert P., Saas P., Seilles E., Aubin F. (2014). Effects of anti-TNF-alpha agents on circulating endothelial-derived and platelet-derived microparticles in psoriasis. Exp. Dermatol..

[291] Ryan C., Leonardi C.L., Krueger J.G., Kimball A.B., Strober B.E., Gordon K.B., Langley R.G., de Lemos J.A., Daoud Y., Blankenship D. (2011). Association between biologic therapies for chronic plaque psoriasis and cardiovascular events: A meta-analysis of randomized controlled trials. JAMA.

[292] Tzellos T., Kyrgidis A., Zouboulis C.C. (2013). Re-evaluation of the risk for major adverse cardiovascular events in patients treated with anti-IL-12/23 biological agents for chronic plaque psoriasis: A meta-analysis of randomized controlled trials. J. Eur. Acad. Dermatol. Venereol. JEADV.

[293] Papp K.A., Griffiths C.E., Gordon K., Lebwohl M., Szapary P.O., Wasfi Y., Chan D., Hsu M.C., Ho V., Ghislain P.D. (2013). Long-term safety of ustekinumab in patients with moderate-to-severe psoriasis: Final results from 5 years of follow-up. Br. J. Dermatol..

[294] Rungapiromnan W., Yiu Z.Z.N., Warren R.B., Griffiths C.E.M., Ashcroft D.M. (2017). Impact of biologic therapies on risk of major adverse cardiovascular events in patients with psoriasis: Systematic review and meta-analysis of randomized controlled trials. Br. J. Dermatol..

[295] Reich K., Langley R.G., Lebwohl M., Szapary P., Guzzo C., Yeilding N., Li S., Hsu M.C., Griffiths C.E. (2011). Cardiovascular safety of ustekinumab in patients with moderate to severe psoriasis: Results of integrated analyses of data from phase II and III clinical studies. Br. J. Dermatol..

[296] Ikonomidis I., Papadavid E., Makavos G., Andreadou I., Varoudi M., Gravanis K., Theodoropoulos K., Pavlidis G., Triantafyllidi H., Moutsatsou P. (2017). Lowering interleukin-12 activity improves myocardial and vascular function compared with tumor necrosis factor-a antagonism or cyclosporine in psoriasis. Circ. Cardiovasc Imaging.

[297] Ng C.Y., Tzeng I.S., Liu S.H., Chang Y.C., Huang Y.H. (2017). Metabolic parameters in psoriatic patients treated with interleukin-12/23 blockade (ustekinumab). J. Dermatol..

[298] Van de Kerkhof P.C., Griffiths C.E., Reich K., Leonardi C.L., Blauvelt A., Tsai T.F., Gong Y., Huang J., Papavassilis C., Fox T. (2016). Secukinumab long-term safety experience: A pooled analysis of 10 phase II and III clinical studies in patients with moderate to severe plaque psoriasis. J. Am. Acad. Dermatol..

[299] Wu D., Hou S.Y., Zhao S., Hou L.X., Jiao T., Xu N.N., Zhang N. (2017). Efficacy and safety of interleukin-17 antagonists in patients with plaque psoriasis: A meta-analysis from phase 3 randomized controlled trials. J. Eur. Acad. Dermatol. Venereol. JEADV.

[300] Torres T., Raposo I., Selores M. (2016). IL-17 blockade in psoriasis: Friend or foe in cardiovascular risk?. Am. J. Clin. Dermatol..

[301] Deeks E.D. (2015). Apremilast: A review in psoriasis and psoriatic arthritis. Drugs.

[302] Crowley J., Thaci D., Joly P., Peris K., Papp K.A., Goncalves J., Day R.M., Chen R., Shah K., Ferrandiz C. (2017). Long-term safety and tolerability of apremilast in patients with psoriasis: Pooled safety analysis for >/= 156 weeks from 2 phase 3, randomized, controlled trials (ESTEEM 1 and 2). J. Am. Acad. Dermatol..

[303] Kavanaugh A., Mease P.J., Gomez-Reino J.J., Adebajo A.O., Wollenhaupt J., Gladman D.D., Lespessailles E., Hall S., Hochfeld M., Hu C. (2014). Treatment of psoriatic arthritis in a phase 3 randomised, placebo-controlled trial with apremilast, an oral phosphodiesterase 4 inhibitor. Ann. Rheum. Dis..

